# Characterization of a novel cold-adapted intracellular serine protease from the extremophile *Planococcus halocryophilus* Or1

**DOI:** 10.3389/fmicb.2023.1121857

**Published:** 2023-02-23

**Authors:** Casper Bøjer Rasmussen, Carsten Scavenius, Ida B. Thøgersen, Seandean Lykke Harwood, Øivind Larsen, Gro Elin Kjaereng Bjerga, Peter Stougaard, Jan J. Enghild, Mariane Schmidt Thøgersen

**Affiliations:** ^1^Department of Molecular Biology and Genetics, Aarhus University, Aarhus, Denmark; ^2^Danish Technological Institute, Aarhus, Denmark; ^3^NORCE Climate and Environment, NORCE Norwegian Research Centre AS, Bergen, Norway; ^4^Department of Environmental Science, Aarhus University, Roskilde, Denmark

**Keywords:** characterization, cold adaptation, protein chemistry, intracellular subtilisin protease, maturation, calcium, Planococcus

## Abstract

The enzymes of microorganisms that live in cold environments must be able to function at ambient temperatures. Cold-adapted enzymes generally have less ordered structures that convey a higher catalytic rate, but at the cost of lower thermodynamic stability. In this study, we characterized P355, a novel intracellular subtilisin protease (ISP) derived from the genome of *Planococcus halocryophilus* Or1, which is a bacterium metabolically active down to −25°C. P355′s stability and activity at varying pH values, temperatures, and salt concentrations, as well as its temperature-dependent kinetics, were determined and compared to an uncharacterized thermophilic ISP (T0099) from *Parageobacillus thermoglucosidasius*, a previously characterized ISP (T0034) from *Planococcus* sp. AW02J18, and Subtilisin Carlsberg (SC). The results showed that P355 was the most heat-labile of these enzymes, closely followed by T0034. P355 and T0034 exhibited catalytic constants (*k*_*cat*_) that were much higher than those of T0099 and SC. Thus, both P355 and T0034 demonstrate the characteristics of the stability-activity trade-off that has been widely observed in cold-adapted proteases.

## Introduction

Microorganisms that thrive at sub-zero temperatures are called psychrophilic or cold-adapted organisms and have been isolated from the high Arctic and Antarctic permafrost (Wilhelm et al., 2012; Goordial and Whyte, 2014; Goordial et al., 2017; Bhatia et al., 2021). One example is *Planococcus halocryophilus* Or1, which grows at −15°C and remains metabolically active at −25°C in water containing 18% (3.08 M) NaCl (Mykytczuk et al., 2012, 2013). These are the lowest temperatures reported for any microbial activity, although optimal temperature for its growth is 25°C and it remains viable at up to 37°C. Therefore, *P. halocryophilus* must be able to express enzymes that can carry out essential metabolic and cellular processes at these sub-zero temperatures. Generally, the enzymes of psychrophilic microorganisms, or cold-adapted enzymes, are thought to be adapted to lower temperatures by increasing their catalytic activity, i.e., the catalytic rate constant (*k*_*cat*_), which usually also results in a higher Michaelis-Menten constant (*K*_*m*_). This is in turn reflected in an increased structural flexibility at the cost of thermodynamic stability (Santiago et al., 2016; Furhan, 2020). This flexibility-stability trade-off is apparent in the enzyme structure of cold-adapted enzymes as (1) larger loop regions, (2) bulkier residues around the active site, (3) a less dense hydrophobic core, and (4) fewer stabilizing salt bridges, among others (Santiago et al., 2016).

Cold-adapted proteins such as proteases are interesting not only from a basic research point of view, but also in the context of applied enzymatic processes (Davail et al., 1994; Lylloff et al., 2016; Pereira et al., 2017; Park et al., 2018). Cold-adapted enzymes have properties that are advantageous in industrial contexts by reducing energy consumption, chemical side-products, and bacterial contaminations. Furthermore, thermal inactivation, as an attractive procedure to denature the enzyme, is easier for cold-adapted enzymes due to their intrinsic thermal lability (Bruno et al., 2019). A number of subtilisins see industrial use, especially in detergents since they are active at alkaline pH (up to pH 12) (Fujinami and Fujisawa, 2010), where proteins become more soluble (Zhang et al., 2015) and show specificity toward aromatic and hydrophobic residues (Groen et al., 1992; Furhan, 2020). All currently available commercial subtilisins are isolated from bacteria within the genus *Bacillus*, ranging from the mesophilic Subtilisin Carlsberg (SC) (e.g., Alcalase™) (Linderstrøm-Lang and Ottesen, 1947; Fasim et al., 2021) to a few cold-adapted subtilisins (Sarmiento et al., 2015). Cold-adapted proteases can be engineered from mesophilic proteases (Wintrode et al., 2000; Tindbaek et al., 2004), where thermal stability may be significantly affected by a single point mutation (Narinx et al., 1997). The demand for cold-adapted enzymes is anticipated to increase (Furhan, 2020); thus, there is a continued need to study naturally occurring cold-adapted enzymes both to understand the mechanisms of cold-adaption and to identify new useful enzymes.

Subtilisins are found both as extracellular subtilisin proteases (ESPs) and intracellular subtilisin proteases (ISPs). ESPs contain a signal peptide that directs their secretion from the cell, as well as a pro-peptide that inhibits activity in their zymogen state and functions as a chaperone during their folding (Zhu et al., 1989; Ohta et al., 1991). The pro-peptide is autoproteolytically removed to produce the active ESP (Ikemura et al., 1987; Ikemura and Inouye, 1988). ISPs do not contain signal peptides but are also synthesized as zymogens. They contain a short (16–25 residues) N-terminal pro-peptide with a LIPY/F motif that is removed during activation in a calcium-dependent manner (Gamble et al., 2011). Calcium additionally stabilizes subtilisins, preventing their auto-proteolysis (Braxton and Wells, 1992) and thermal inactivation (Voordouw et al., 1976; Veltman et al., 1998). ISPs constitute most of the intracellular degradome in *Bacillus subtilis* (Orrego et al., 1973; Burnett et al., 1986) and share 40–50% amino acid sequence identity with ESPs (Vévodová et al., 2010). Gamble et al. (2011) proposed a maturation model of the ISP from *Bacillus clausii* where a small fraction of pro-ISP adopts an “open” conformation where the pro-peptide is transiently dislocated from the active site. They proposed that calcium may facilitate this conformation by binding at the S1 site, thus replacing water as the ligand to Glu20 with sodium, stabilizing the “open” conformation, which can then process zymogenic ISP to active ISP in an intermolecular manner.

This study aimed to characterize new cold-active proteases to learn more about cold-adaptation in enzymes to harness their potential for industrial applications. The novel ISP gene encoding P355 was mined from the genome of the cold-active permafrost bacterium *P. halocryophilus* Or1 (Mykytczuk et al., 2012, 2013) and was expressed in *Escherichia coli* as a C-terminally His-tagged enzyme. We performed a thorough characterization of P355 and compared this with another uncharacterized, putatively thermophilic ISP (T0099), as well as two previously characterized serine proteases: the putative mesophilic ISP (T0034) from *Planococcus* sp. AW02J18 (Bjerga et al., 2018) and the mesophilic ESP Subtilisin Carlsberg, as an industrial reference protease.

## Materials and methods

### Materials

All chemicals were purchased from Merck KGaA (Darmstadt, Germany) with purity >98% unless otherwise noted. A commercial formulation of the ESP Subtilisin Carlsberg (SC) from *Bacillus licheniformis*, type VIII, (Merck, cat. no. P5380) was included in activity assays.

### Identification of an intracellular subtilisin protease

The DNA sequence encoding a subtilisin-like intracellular protease was identified by sequence-based mining of the cryophilic permafrost bacterium *P. halocryophilus* Or1 (GenBank acc. no. ANBV00000000) (Mykytczuk et al., 2012). Isolation and sequencing of genomic DNA followed by assembly of the genome were carried out in a different study (Mykytczuk et al., 2013). Annotation was carried out using the Rapid Annotation Technology (RAST) (Aziz et al., 2008; Brettin et al., 2015) and potential subtilisin-coding ORFs were identified in Geneious (ver. 2020.0.4). Subtilisin candidates were analyzed for subtilisin domains and leader sequences using InterProScan (Jones et al., 2014) and SignalP 6.0 (Nielsen et al., 1997; Teufel et al., 2022), respectively. Three potential subtilisin genes were identified, but only one was active in pre-liminary protease assays, and became the target for this study: The codon-optimized DNA sequence of the identified active candidate, ISP P355, has been deposited in GenBank with accession no. OP748402. For comparative studies, we used the previously reported ISP from *Planococcus* sp. AW02J18 (Bjerga et al., 2018) (T0034, Genbank accession no. MG786190) and an uncharacterized ISP from *Parageobacillus thermoglucosidasius* (T0099; Genbank accession no. WP_003251350), which is known to be a thermophilic Gram-positive bacterium (Aliyu et al., 2016). Similarities of protein sequences were analyzed in Geneious Prime (v. 2022.2.2). Distances were calculated by pairwise alignment using Clustal Omega 1.2.2.

### Sub-cloning of the ISP gene to expression vectors

For recombinant cloning and enzyme expression, we used a previously developed screening procedure for subtilisin-like proteases (Bjerga et al., 2016). The P355 and T0099 ISP protein sequences were used as templates for gene synthesis (GenScript), and the gene was codon optimized for expression in *E. coli*. The *isp* gene was synthesized with flanking *Sap*I restriction sites and produced in a *Sap*I-free pUC57 vector (kanamycin resistant). The *isp* gene was sub-cloned from the delivery vector to the p12 FX-cloning vector (ampicillin resistant) used in Bjerga et al. (2016) to allow a fusion of a C-terminal hexahistidine tag for downstream purification. T0034 was prepared in the exact same manner, as outlined in Bjerga et al. (2018). The constructs were transformed into chemically competent *E. coli* MC1061 for expression.

### Expression and protein purification

Intracellular subtilisin proteases with C-terminal His-tags were expressed from *E. coli*. Cells were cultured on lysogeny broth (LB) agar plates containing 100 μg/mL ampicillin overnight at 37°C. A single colony was inoculated in 10 mL LB with 100 μg/mL ampicillin and incubated overnight at 37°C, 200 rpm. Five mL overnight culture was transferred to 100 mL LB with 100 μg/mL ampicillin and incubated for approx. 5 h at 37°C with 200 rpm until an OD_600_ of 0.5–0.7 before 1 mL sterile 10% L-arabinose was added for induction. The incubation temperature was then adjusted to 20°C, and cultures were incubated overnight at 200 rpm. Cells were harvested by centrifuged at 1000 × *g* for 10 min and resuspended in 40 mL buffer A1 (500 mM NaCl, 30 mM imidazole, 20 mM sodium phosphate, pH 7.4). Cells were lysed by sonication (Qsonica) using a 12 mm probe in ice water using a cycle of 1-s sonication and 1-s pause for 6 min in total at 13% amplitude. The lysate was centrifuged at 22,000 × *g* at 4°C to remove cell debris. The following steps were performed at room temperature (RT), but the protein was kept on ice whenever possible. A 5 mL HisTrap high performance (HP) column (GE Healthcare) was equilibrated in buffer A1 using an ÄKTA purifier system (GE Healthcare) before lysate was loaded. The column was then washed with 5 column volumes of buffer A1 followed by 5 column volumes of 10% B1 where 100% B1 is 500 mM imidazole, 20 mM sodium phosphate, pH 7.4. Finally, the protein was eluted using 100% B1. Additional impurities were removed by applying eluate from the HisTrap HP column to a 5 mL HiTrap Q high performance (GE Healthcare) pre-equilibrated in buffer A2 (10 mM Tris, pH 8). The column was washed with 5 column volumes of buffer A2 before a linear gradient from 0 to 1 M NaCl was applied. A flow of 5 mL/min was used throughout the FPLC-steps and collected into 1 mL fractions. Purity (Supplementary Figure 1) was assessed by SDS-PAGE (see Section “SDS-PAGE”), and fractions were tested for protease activity (see Section “Protease activity assays”). The yield was typically 5–20 mg protein pr. L medium. SC was dissolved in MQ (2 or 3.74 μg/μL) with no further purification. Protein concentrations were determined by absorbance at 280 nm (path length = 1 cm) and using the extension coefficients calculated by ProtParam (Gasteiger et al., 2005) and stored at −20°C.

### SDS-PAGE

Proteins for SDS-PAGE were denatured by heating to 95°C for 5 min in sample buffer (1% SDS, 5–15 mM DTT, bromphenol blue) and separated using the glycine/2-amino-2-methyl-1,3-propanediol/HCl (ammediol) system in 5–15 or 10–15% acrylamide gradient gels (Bury, 1981) casted in-house (10 × 10 × 0.15 cm). The gels were stained with Coomassie Brilliant Blue.

### Protease maturation

Proteases P355, T0034, T0099, and SC (1.9, 2, 2, and 2.5 μM, respectively) were incubated in maturation buffer (25 mM CaCl_2_, 0.1% (w/w) Triton X-100 (1.7 mM), 100 mM NaCl, and 100 mM glycine, pH 9.5). The proteases were incubated for 10, 30, 60, 90, 120, and 180 min at RT before SDS-PAGE and activity measurement. For SDS-PAGE, 20 μL of each protease solution, each containing approximately 1 μg of the enzyme, was mixed with 12 μL sample buffer (see Section “SDS-PAGE”) and denatured at 95°C for 5 min. Additionally, 10 μL was tested for activity where the final protease concentration of P355, T0034, T0099, and SC were 1.63, 1.75, 2.06, and 0.94 nM, respectively (see Section “Protease activity assay”).

### N-terminal sequencing

Samples destined for Edman degradation were separated by SDS-PAGE as described above, except that the samples were denatured at 80°C for 5 min. The unstained gel was equilibrated in blotting buffer (20% ethanol (v/v) and 0.1 M CAPS pH 11) and electrotransferred to polyvinylidene difluoride (PVDF) membranes (Matsudaira, 1987). The PVDF membranes were stained with Coomassie Brilliant Blue in 50% (v/v) methanol and destained with 50% (v/v) methanol. Protein bands were excised with a clean scalpel and applied to trifluoroacetic acid-treated glass fiber membranes. SC was not electroblotted. Instead, Edman degradation was done directly on a SC stock (2 μg/μL in MQ). The automated Edman degradation was performed in a PPSQ-31B protein sequencer (Shimadzu Biotech) with in-line phenylthiohydantoin analysis using an LC-20AT HPLC system and recorded by the Shimadzu PPSQ-31B software. The sequence was determined through manual inspection of the UV 269 nm chromatograms.

### Protease activity assays

#### Preparation for activity assays and reaction conditions

Matured protease stocks were prepared by incubating for 2 h at RT in maturation buffer (25 mM CaCl_2_, 0.1% (w/w) Triton X-100, 100 mM NaCl, and 100 mM glycine, pH 9.5) as described in “Protease maturation” to mature the proteases before activity assays except for the “Calcium titration” experiment. Matured proteases were kept on ice before experiments. Protease activity was measured in a FLOUstar Omega plate reader (Thermo-Fischer*™*) in half-area plates (Corning^®^) at 410 nm and at RT using 0.2 mM N-Succinyl-Ala-Ala-Pro-Phe p-nitroanilide (AAPF) as substrate. A 100 mM AAPF stock was made in 100% dimethylsulfoxide (DMSO). The final reaction volume was 100 μL containing 0.2% DMSO and 0.2 mM AAPF.

All protease activity assays contained reaction buffer (25 mM CaCl_2_, 100 mM glycine, pH 9.5, 0.1% (w/w) Triton X-100, and 100 mM NaCl) unless otherwise stated and carried out at RT. The final protease concentration was approximately 1–4 nM in every activity assay described below unless otherwise stated. This amount of protease produced linear curves with *r*^2^ ≥ 0.99 based on linear regression of the initial reaction (0–4 min). Every incubation was carried out at RT unless otherwise stated and activity measurement was done in at least technical triplicates.

#### Calcium titration

Matured protease incubated in reaction buffer containing 0–62.5 mM CaCl_2_ for 30 min before AAPF was added. pH was adjusted to pH 9.5 except for T0099, which was incubated at pH 7.5.

#### Inhibition

Inhibition of activity was assessed by testing the effect of EDTA and Pefabloc^®^ on protease activity. For the EDTA assay, 2 μL protease was added to the reaction buffer with no added CaCl_2_ (protease solution contributed with 63 μM CaCl_2_ from the maturation step) and 0–1 mM EDTA and incubated for 60 min. For the Pefabloc^®^ assay, 5 μL matured protease was added to the reaction buffer with calcium in 0–5 mM Pefabloc^®^. Tris at pH 7.5 was used in the reaction buffer to reduce the auto-hydrolysis of Pefabloc^®^. The protease was incubated with Pefabloc for 2.5 h RT.

#### pH optimum

Matured protease was added to a reaction buffer containing the following buffers to determine the pH optimum: glycine at pH 2.5–3.5; sodium acetate at pH 4–5.5; MES at pH 5.5–6.5; HEPES at 7–7.5; Tris at pH 7.5–8.5; and glycine at pH 9–10.5. Buffer concentrations were 0.1 M.

#### Temperature optimum

Half-area plates containing reaction buffer were adjusted to 25 or 45°C on an Eppendorf ThermoMixer C. Water filled the gaps between the wells to stabilize the temperature resulting in low standard deviations along with *r*^2^ > 0.99. CAPS at pH 9.7 was used as a buffer instead of glycine as growth occurred in prolonged storage of glycine buffer (0.4 M glycine). The effect of temperature on pK_*a*_ of CAPS was compensated by using d(pK_*a*_)/dT = −0.009. AAPF stock (100 mM) was diluted with temperature-adjusted water and incubated for 10 min. at either 25 or 45°C in the plate. Finally, 5 μL matured protease was added, and activity was measured immediately.

#### pH stability

Five μL matured protease was incubated in 95 μL incubation solution [0.1% Triton X-100, 100 mM NaCl, and 100 mM buffer (glycine for pH 2.5 and 3.5, sodium acetate for pH 4.5 and 5.5, MES for pH 6.5, Tris for pH 7.5 and 8.5, and glycine for pH 9.5 and 10.5)]. Five μL of the incubation solution was aspirated after 10, 30, 60, 120, and 180 min, and activity was measured in reaction buffer with a final volume of 100 μL. The pH was adjusted to pH 9.5 by the reaction buffer for all measurements. Calcium was omitted in the incubations and reaction steps due to precipitation, except for the carry-over calcium from the stock solutions.

#### Temperature stability

Matured proteases were incubated in maturation buffer at different temperatures, and samples were aspirated at different time intervals: 0°C (ice-water) for 24, 48, and 120 h; 25°C for 24, 48, and 120; 45°C for 10, 20, 30 min, 1–4, 24, and 48 h; and 65°C for 10, 20, 30 min, 1–4, 24, and 48 h The remaining activity was measured as described above and normalized to the corresponding matured protease, which had incubated for 0 h at the given temperatures.

#### Salt titration

Five μL matured protease was added to a reaction buffer containing titrated NaCl (1–1000 mM), KCl (1–1000 mM), urea (4–4000 mM), or guanidine hydrochloride (Gnd) (4–4000 mM) and incubated for 30 min before the activity was measured. The activity was normalized to the lowest salt concentration: 0.98 mM for NaCl and KCl and 3.91 mM for urea and Gnd.

#### Michaelis-Menten kinetics

Michaelis-Menten kinetics were determined by measuring the activity at different substrate concentrations ranging from 0.03 to 4 mM (using a 400 mM AAPF stock) in reaction buffer at 25 and 45°C. Protease concentrations were 0.94, 10.02, 4.06, and 2.37 nM for P355, T0034, T0099, and SC, respectively. The concentration of T0034 was lowered to 1.02 nM at 45°C. Initial rate was determined by linear regression (*r*^2^ > 0.99). Absorbance was converted to mM by the slope of the linear standard curve (*r*^2^ > 0.99), where the substrate had been hydrolyzed completely. Enzymatic activity was defined as the turnover rate of millimolar substrate pr. hour (mmol/h). The kinetics constants were derived with R (v. 4.0.5) using the add-on package *drc* (Ritz et al., 2016) by non-linear fit (least squares estimation) with a confidence interval of 0.99.

#### Casein digest

Proteolysis of resorufin-labeled casein was assessed by incubating 3 nM matured P355, T0034, and SC, and 60 nM matured T0099 with 0.5 μg/μL resorufin-labeled casein in 100 mM or 1000 mM NaCl. A reaction was quenched every 2.5 min with 2% (v/v) TCA and incubated for 10 min at 37°C, precipitating undigested casein. The solution was filtered using a MultiScreen Solvinert Filter plate (Millipore) by centrifugation at 500 × *g* for 5 min. and filtrate was collected in a Corning^®^ 96 well plate. The filtrate was adjusted to pH 8.8 using 3.9 M Tris, and absorbance was measured at 574 nm. The reaction rate was linear within the first 10 min (*r*^2^ > 0.99). Proteolytic activity against casein was defined as A × h^–1^ × nM^–1^ (the slope divided with the protease concentration).

### raBSA digest in ice-water

The BSA was dissolved in 8 M urea (0.27 mM), and the disulfide bridges were reduced using 10 mM DTT for 60 min at RT. Iodoacetamide (30 mM) was added, and the sample was incubated for 60 min at RT in the dark before 35 mM DTT was added to quench the reaction. The reduced and alkylated BSA (raBSA) was dialyzed into 10 mM Tris pH 8 so that [urea] <10 mM and stored at −20°C. Titrated matured protease (0.4–100 nM) incubated with 3.75 μg raBSA (1.88 μM) in reaction buffer containing 100 or 1000 mM NaCl for 2 h in ice-water. Controls were raBSA without protease and protease (highest concentration) without raBSA. The pH shift in the reaction buffer due to the lower temperature was considered by using d(pKa)/dT = −0.025. The reaction was terminated by adding SDS-PAGE sample buffer, DTT (see Section “SDS-PAGE”) along with 42 mM EDTA and heating to 95°C for 10 min. Digestion patterns were examined with SDS-PAGE, and the density of the raBSA band was measured using Gel Doc™ EZ imager (Biorad) software.

### Cleavage sites in BSA and micro purification for mass spectrometry

A total of 3.8 μg raBSA (BSA digest in ice water) was loaded onto an SDS-PAGE to separate intact raBSA from impurities and nicked raBSA. The raBSA band was cut out and washed twice with 500 μL H_2_O followed by two times incubation of 15 min with 50 μL 50% acetonitrile. Then, 50 μL of 100% acetonitrile was added and incubated for 15 min before the reaction buffer was added to a 1:1 ratio. The liquid was removed after 15 min and lyophilized until the gel plug was dry. Matured protease (600 nM) was added to the dried plugs and incubated for 10 min. Thirty μL reaction buffer was added and incubated for 1.5 h. Triton X-100 was excluded as detergents are not compatible with MS. All incubation was performed at RT except protease digestions, which were allowed to proceed at 37°C. The reaction was quenched with 7.1% formic acid. The resulting peptides from protease digestion were micro-purified using C18 column material (Empore™) in P10 pipette tips (Sarstedt). The column material was activated with solvent B (99.9% acetonitrile and 0.1% formic acid) and then equilibrated with solvent A (0.1% formic acid). Peptides were then loaded and washed in solvent A before being eluted with 70% solvent B and dried for MS. The experiment was carried out in triplicates.

### LC-MS/MS and data processing

Nano LC-MS/MS was carried out on an Orbitrap Eclipse Tribrid mass spectrometer connected online to an EASY nanoLC 1200 (both instruments were from Thermo Fisher Scientific). Samples were dissolved in solvent A, desalted on a ReproSil-Pur C18-AQ trap column (2 cm × 100-μm inner diameter packed in-house with 3 μm resin (Dr. Marisch GmbH, Ammerbuch-Entringen, Germany). The raBSA-peptides from the protease digestion were eluted and separated on a 15-cm analytical column (75 μm inner diameter) packed in-house with ReproSil-Pur C18-AQ 3 μm resin (Dr. Marisch GmbH, Ammerbuch-Entringen, Germany). A flow rate of 250 nL/min was used to elute the peptides with a 50-min gradient from 5 to 35% solvent B.

The generated raw files were converted to Mascot generic format. Data was searched in Mascot (version 2.5.0) against the SwissProt Data base (565,928 sequences composed of 204,173,280 residues). The entire SwissProt database was used as a decoy database (random sequence). The following search parameters were (1) ‘none’ as the protease, (2) minimum 6 residues in a peptide, (3) precursor mass deviation of maximum 10 ppm, (4) fragment mass deviation of maximum 0.02 Da, (5) dynamic modification with oxidation on Met, and (6) fixed modification with alkylation (carbamidomethyl) on Cys. The false discovery rate was set to 1% before data was exported to MS Data Miner (Dyrlund et al., 2012). Peptides having a score lower than the significant score provided by the Mascot search were discarded.

### R programming and statistics

Data analysis and figures were made in R (version 4.0.5) (R Core Team, 2021) coupled with RStudio Team (2020). The following packages were employed for general data analysis: *tidyverse* (Wickham et al., 2019), *tagger* (Campitelli, 2022), *lemon* (McKinnon Edwards, 2020), *readxl* (Wickham and Bryan, 2019), and *ggExtra* (Attali and Baker, 2019). Additional packages used for a specific experiment are cited in the given method description. All error bars show the standard deviation (SD) of triplicates or more.

## Results

### Sequence alignment of ESP and ISP

The well-known, mesophilic ESP Subtilisin Carlsberg (SC) from *Bacillus licheniformis* (Linderstrøm-Lang and Ottesen, 1947) was compared to two uncharacterized ISP proteases, the putative cold-adapted P355 and the putative thermophilic T0099 from *Parageobacillus thermoglucosidasius*, and to a previously characterized ISP protease, T0034 from *Planococcus* sp. AW02J18. The latter origin from a marine bacterium isolated from coastal waters near Lofoten in Norway (Bjerga et al., 2018). P355 is presumed to be cold-adapted as it is from the genome sequence of the extremophile *P. halocryophilus* Or1 identified in Arctic permafrost (Mykytczuk et al., 2013). T0099 is presumed to be thermophilic as it originates from the genome of *Parageobacillus thermoglucosidasius*, known to thrive at temperatures up to 68°C (Aliyu et al., 2016). To the authors’ knowledge, no thermophilic ISP has been described.

Pairwise alignment of full ISP protein sequences, including pre-sequences, revealed that the closest related protein sequences to T0099 were an ISP from *Paenibacillus polymyxa* (acc. no. P29139) (64% amino acid similarity) (Takekawa et al., 1991) as well as the major ISP from *B. subtilis* subsp. *subtilis* str. 168 (acc. no. P11018) (64% amino acid similarity) (Koide et al., 1986). P355 shared 72% amino acid sequence identity to the characterized ISP from *Planococcus* sp. AW02J18, henceforth referred to as T0034 (Bjerga et al., 2018), which is included in this study for comparison. As expected, analysis using SignalP showed that neither of the ISP sequences were predicted to have an N-terminal signal peptide for extracellular export. Similar to T0034 (Bjerga et al., 2018), the novel ISPs T0099 and P355 contained a short pro-peptide with a conserved LIPY/F-sequence at the N-terminus (T0099: LIPF, P355: LIPY) (Supplementary Figure 2; Vévodová et al., 2010; Gamble et al., 2011; Bjerga et al., 2018). The catalytic domains of ESPs and ISPs are homologous, with all three residues involved in the catalytic tried being conserved, and both members are part of the Subtilisin Peptidase S8 family as classified by Pfam. The major difference between ISPs and ESPs lies in the absence of signal sequences in the ISPs and the presence of a shorter pro-sequence for the regulation of catalytic activity (Gamble et al., 2011).

### Calcium induces the activation of P355, T0034, and T0099

**FIGURE 1:**
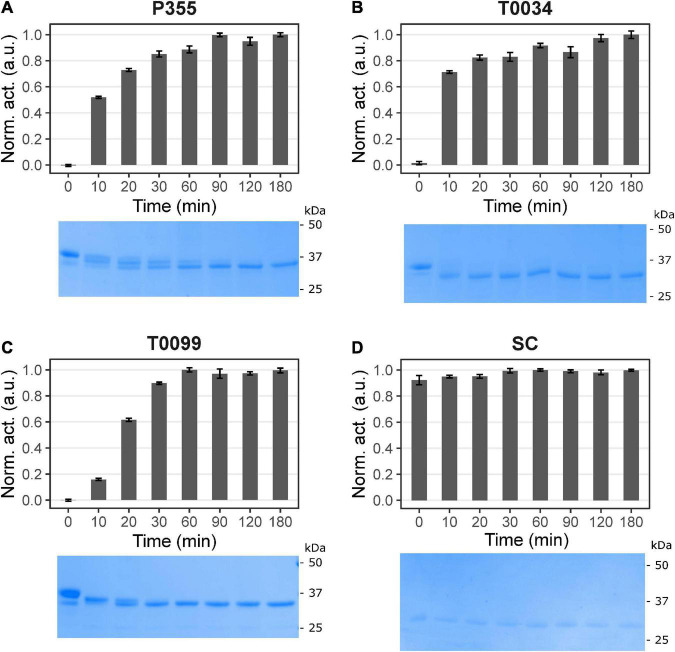
Time course maturation of the proteases. Maturation was started by incubating in 100 mM NaCl, 25 mM CaCl_2_, 100 mM glycine pH 9.5, and 0.1% Triton X-100. The maturation process was monitored with activity assay using 0.2 mM N-Succinyl-Ala-Ala-Pro-Phe p-nitroanilide (AAPF) and SDS-PAGE at different time intervals in minutes: after 0, 10, 20, 30, 60, 120, and 180 min incubation. The sample at 0 min had not received CaCl_2_. The activity was normalized to the highest activity measured for the given proteases and plotted against the conditions. No activity and proteolytic processing occurred for panel **(A)** P355, **(B)** T0034, and **(C)** T0099 without calcium. Calcium induced activity and proteolytic processing, i.e., maturation. Maturation was complete after 90, 120, and 60 min for P355, T0034, and T0099, respectively, judged by the activity plateau and no further band migration. Subtilisin Carlsberg (SC) **(D)** was unaffected throughout the incubation. Error bars show standard deviation (*n* = 3). Note that the figures are cropped. See Supplementary Figure 4 for uncropped images.

Subtilisins are zymogens, i.e., they become proteolytically active through a maturation process where the pro-peptide at the N-terminal is removed (Neurath and Walsh, 1976). This process can be calcium-dependent (Gamble et al., 2011; Bjerga et al., 2018) as well as pH-dependent (Bjerga et al., 2018), and can be a relatively slow process (Gamble et al., 2011). For this study, the three ISPs, P355, T0034, and T0099, were recombinantly expressed in *E. coli* with their pro-peptide sequences intact. Histidine tags fused to the C-terminal allowed downstream purification by nickel affinity and anion exchange chromatography in the absence of calcium to avoid maturation (Bjerga et al., 2018). A commercial formulation of ESP, SC, was purchased, and used as a reference in the experiments. To investigate the activation of the ISPs, we determined their proteolytic activity toward a colorimetric peptide [N-Succinyl-Ala-Ala-Pro-Phe p-nitroanilide (AAPF)] as a model substrate after incubation with a titration series of calcium. The proteolytic activity of the three recombinant ISPs showed dependence on CaCl_2_ ranging from 0.2 to 62.5 mM CaCl_2_ (Supplementary Figure 3) with no activity observed at 0 mM CaCl_2_. The ESP was independent of CaCl_2_, i.e., activity was observed at 0 mM CaCl_2_ with a slight decrease in activity in the same CaCl_2_ concentration range. Next, we investigated the maturation of the ISPs over time after initiation using 25 mM CaCl_2_, as assessed by AAPF activity and SDS-PAGE (Figure 1). All three ISPs in their zymogenic form were inactive and unprocessed before 25 mM CaCl_2_ was added (Figures 1A–C). No processing or activity was observed even after 180 min incubation without calcium (Supplementary Figures 4E, F). In the presence of 25 mM CaCl_2_, the activity of the ISPs increased over time, concurrent with a corresponding change in the migration of bands visualized by SDS-PAGE from the full-length zymogen to the truncated active protease. N-terminal protein sequencing of zymogens, prepared in the absence of calcium treatment, showed that zymogens lacked the first amino acid (Met). Active P355, T0034, and T0099, i.e., 2 h incubation with 25 mM CaCl_2_ at pH 9.5, lacked an additional 9, 15, and 16 N-terminal residues, respectively (Supplementary Figure 2), demonstrating that the pro-peptide and its LIPY/F motif were removed in all three active ISPs. Additional intermediate bands were visible for P355 (Figure 1A) and T0099 (Figure 1C) in SDS-PAGE analysis of the maturation process, indicating that their activation may occur in distinct steps. The activity reached a maximum and the conversion to the active protease was complete after 120, 120, and 60 min. for P355, T0034, and T0099, respectively. SC showed no change in activity or processing with or without calcium incubation (Figure 1D and Supplementary Figures 4E, F) thus verifying that its pro-peptide was fully removed during commercial production and formulation. Based on these data, we defined the conditions that produce matured and active proteases and henceforth, the three ISPs are referred to simply as proteases. Based on these data, ISPs were routinely activated for 2 h in the presence of calcium prior to their use in subsequent assays. The relative number of active sites between the subtilisins were estimated by α-2-macroglobulin titration (Supplementary Figures 5–9). By a rough estimation, P355, T0034, and SC contained relatively the same number of active sites, while T0099 contained approximately half as many (Supplementary Figure 8) indicating less correctly folded protein. The metal chelator EDTA inhibited the three ISPs, but SC was largely unaffected (Supplementary Figure 10A). The commercial serine protease specific inhibitor Pefabloc^®^ blocked activity from all proteases (Supplementary Figure 10B).

### Effect of pH and temperature on activity and stability

**FIGURE 2:**
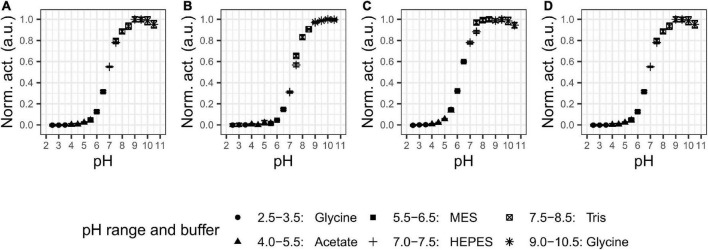
pH optima. Activity was assessed with 0.2 mM N-Succinyl-Ala-Ala-Pro-Phe p-nitroanilide (AAPF) for matured proteases under different pH values. The legend shows the buffers used in the different pH ranges. Every protease showed activity the alkaline range with optimal pH at **(A)** 9–9.5 for P355; **(B)** 10 for T0034; **(C)** 8–9.5 for T0099; and **(D)** 9–9.5 for Subtilisin Carlsberg (SC). Activity was normalized to the highest activity for the given protease. Error bars show standard deviation (*n* = 3). Note that some error bars are within the marker.

**FIGURE 3:**
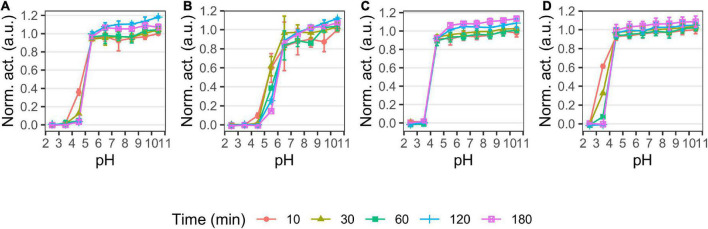
pH stability of P355 **(A)**, T0034 **(B)**, T0099 **(C)**, and Subtilisin Carlsberg (SC) **(D)**. Matured proteases were incubated in different pH values and activity was measured after 10, 30, 60, 120, and 180 min at pH 9.5 with 0.2 mM N-Succinyl-Ala-Ala-Pro-Phe p-nitroanilide (AAPF) at room temperature (RT). Activity was normalized to the highest activity for the given protease at 10 min. The protease activity did not decline over time in the alkaline range, but activity in the acidic range decreased in the following order: SC > T0099 > P355 > T0034. Error bars show standard deviation (*n* = 3).

We examined the pH and temperature profiles, in part to determine the optimal experimental conditions, but also to establish temperature optima and stability, which is typically lower for cold-adapted enzymes compared to meso- and thermophilic enzymes (Furhan, 2020). All proteases showed activity in a broad alkaline range (Figure 2) with optima at pH 9–9.5 for P355, pH 10 for T0034, pH 8–9.5 for T0099, and pH 9–9.5 for SC. The proteases were stable at alkaline pH and restored full activity after 3 h (Figure 3). There was a tendency for activities to slightly increase after 1 h incubation in alkaline conditions. The stability declined toward the acidic range for all enzymes, where no activity remained after 3 h pre-incubations at pH ≤ 3.5 for any of the proteases (Figures 2, 3). The stabilities ranked in the following order from most to least stable based on the remaining activity at pH 3.5–5.5: SC > T0099 > P355 > T0034.

**FIGURE 4:**
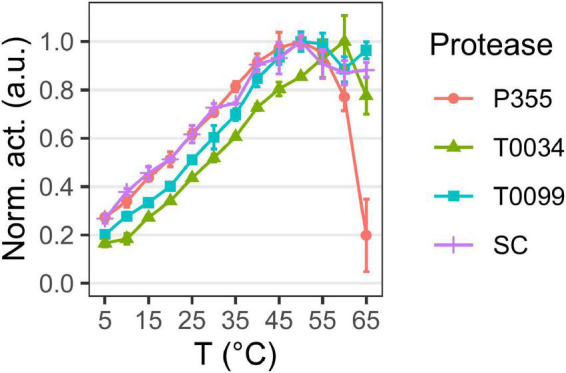
Temperature optima. Matured protease activity was measured under different temperatures using 0.2 mM N-Succinyl-Ala-Ala-Pro-Phe p-nitroanilide (AAPF). Activity was measured at pH 9.7 using 0.1 M CAPS. Changes in pK_*a*_ with temperature were compensated with d(pKa)/dT = –0.009 in buffer preparation. Activity was normalized to the highest activity for the given protease. Matured P355, T0034, and Subtilisin Carlsberg (SC) have a temperature optimum at 50°C, while matured T0034 has a temperature optimum at 60°C. Matured P355 showed less activity at the highest temperatures compared to the others. Error bars show standard deviation (*n* = 3).

**FIGURE 5:**
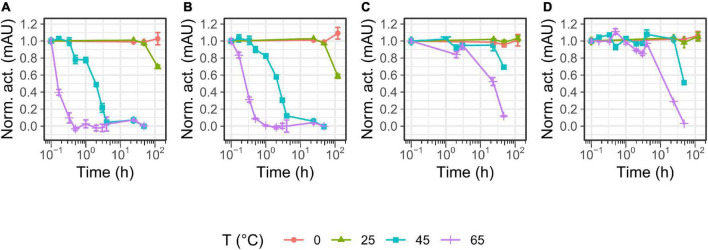
Temperature stability of P355 **(A)**, T0034 **(B)**, T0099 **(C)**, and Subtilisin Carlsberg (SC) **(D)**. The matured protease incubated in maturation buffer [25 mM CaCl2, 0.1% (w/w) Triton X-100, 100 mM NaCl, and 100 mM glycine pH 9.5] at 0 (ice-water), 25, 45, and 65°C ranging between 10 min and 120 hr. Remaining activity was assessed with 0.2 mM N-Succinyl-Ala-Ala-Pro-Phe p-nitroanilide (AAPF) and normalized to 0 h. Note that 10^– 1^ h is defined here as 0 h and was only exposed to room temperature (RT). Every protease was stable for 120 h at 0°C. P355 lost all activity after 30 min at 65°C, while T0034 lost all activity after 1 h. T0099 was the most stable with approximately 10% remaining after 48 h incubation at 65°C. Activity was almost gone after 48 h for SC. Error bars show standard deviation (*n* = 3).

The temperature optimum (Figure 4) of the proteases was determined to be 50°C for P355, T0099, and SC, and 60°C for T0034. The proteases were active at 65°C, but P355 activity dropped to 20% of its maximum activity. P355 and SC showed roughly 50% higher activity at 5°C than T0034 and T0099. Thermal stability was determined by measuring the remaining activity after incubation at different temperatures. Every protease retained 100% activity at 0°C for the duration of the experiment (120 h). While T0099 and SC also remained 100% active after 120 h at 25°C, P355 and T0034 had lost 30–40% of their activity (Figure 5). P355 and T0034 lost all activity after 48 h at 45°C, where T0099 and SC still retained approximately 70 and 50% activity, respectively. At 65°C, P355 and T0034 lost their activities after 30 and 60 min, respectively, while T0099 and SC restored 11% and 3% activity after 48 h, respectively.

Overall, the pH profiles indicate active and stable proteases in the alkaline range. P355 and T0034 have lower thermal stability than T0099 and SC, consistent with the expectations for cold-adapted proteases. However, the temperature optima were similar, although P355 was relatively less active at 65°C.

### Salt titrations and casein digest

**FIGURE 6:**
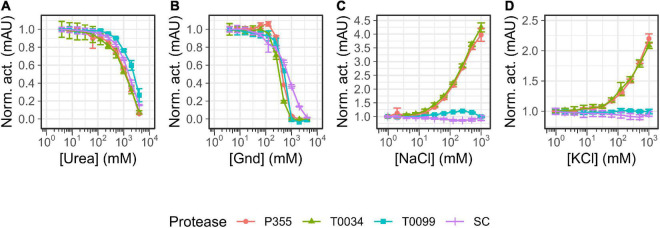
Salt titrations. Matured protease was titrated with 1–1000 mM NaCl or KCl or 4–4000 mM urea or guanidium chloride (Gnd) and incubated for 30 min at room temperature (RT) before activity was measured with 0.2 mM N-Succinyl-Ala-Ala-Pro-Phe p-nitroanilide (AAPF). **(A)** Urea titration lowers protease activity with T0099 being the most resilient. **(B)** Gnd decreases protease activity where Subtilisin Carlsberg (SC) was the most resilient. One M Gnd effectively inhibited protease activity for P355, T0034, and T0099, while 4 M Gnd was needed to abolish SC activity. **(C)** NaCl enhanced P355 and T0034 activity with increasing salt concentration, while T0099 and SC were largely unaffected in comparison. **(D)** KCl also enhanced activity of P355 and T0034 and as with NaCl, T0099 and SC were largely unaffected. Data were normalized to the lowest salt concentration in question. Error bars show standard deviation (*n* = 3).

Chemical stability was also investigated, as cold-adapted proteases are usually more susceptible to chemical denaturation by guanidium chloride (Gnd) and urea than their meso- and thermophilic counterparts (D’Amico et al., 2003). Overall, increasing concentrations of urea (Figure 6A) and Gnd (Figure 6B) reduced the activity of all proteases. P355 and T0034 lost more activity at lower urea concentrations relative to SC and T0099, where T0099 retained most activity. Titration with Gnd showed a different pattern: while the curves of P355 and T0034 are to the left of T0099 and SC in the urea titration (Figure 6A), the activity profiles cross each other with increasing Gnd (Figure 6B). At 0.5 M Gnd P355 and T0034 show the least activity followed by T0099 and SC. These data are in line with prior data (D’Amico et al., 2003), which indicated that cold-adapted proteases are more prone to chemical denaturation.

**FIGURE 7:**
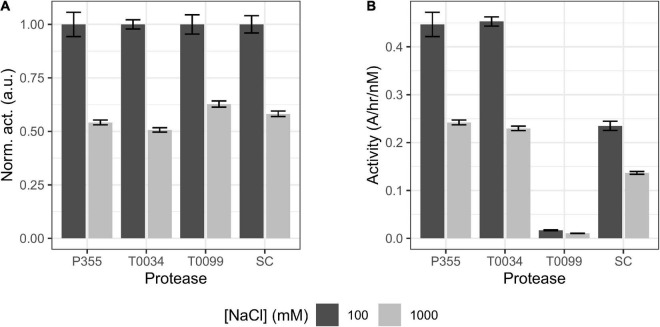
Bar plot of casein digest. The ability of matured proteases to digest casein in the presence of 100 or 1000 mM NaCl was examined. P355, T0034, and Subtilisin Carlsberg (SC) concentrations were 3 nM, while T0099 concentration was 60 nM as no activity was observed at 3 nM (data not shown). **(A)** Activity was normalized to the maximum activity of the respective protease. Increasing [NaCl] from 0.1 to 1 M reduced the hydrolysis of casein of every protease by 40–50%. **(B)** The same data set, but with activity normalized to matured protease concentration (nM). P355 and T0034 showed equal activity, while T0099 was poor in comparison. SC showed intermediate efficiency. Error bars show standard deviation (*n* = 3). Every regression had *r*^2^ > 0.99. Casein did not precipitate in 1 M NaCl (data not shown).

Microorganisms that thrive in subzero degrees are usually also halophilic or halotolerant as salt lowers the freezing point of water (Mykytczuk et al., 2013). Thus, the effect of ionic strength on the catalytic activity using NaCl or KCl was investigated. Increasing concentrations of NaCl and KCl enhanced the activity of P355 and T0034 toward the AAPF peptide substrate. 1M NaCl increased their activities approximately 4-fold (Figure 6C), whereas 1M KCl increased P355′s and T0034′s activity by approximately 2-fold (Figure 6D). We investigated whether the enhancement of activity with increasing NaCl also occurred for other types of substrates, e.g., larger substrates, as these are also potential substrates. For this purpose, we used resorufin labeled casein (20 kDa). When casein is used as a substrate instead of the AAPF peptide, activity decreased for all proteases when NaCl concentration increased from 100 to 1000 mM (Figure 7A). Thus, it seems that the effect of NaCl is substrate-dependent. Normalizing the activity to the protein concentration shows that P355 and T0034 hydrolyzed casein most efficiently followed by SC and finally T0099 (Figure 7B).

### P355 and T0034 have higher catalytic constants compared to T0099 and SC

Cold-adapted enzymes typically have a higher catalytic constant (*k*_*cat*_) than their meso- and thermophilic homologs to compensate for the general decrease in activity at low temperatures (Feller et al., 1992). This was examined by performing Michaelis-Menten kinetics at 25 and 45°C. Judging from the fitted curves (Supplementary Figure 11) and the residuals of the fitted curves to the experimental data points (Supplementary Figure 12) and the low deviations of the fitting parameters (Supplementary Table 1), the proteases mostly follow Michaelis-Menten kinetics. However, T0099 deviates from Michaelis-Menten kinetics at high substrate concentrations (3 and 4 mM), which is also reflected in the residual plot (Supplementary Figures 12E, F). P355 and T0034 have higher *k*_*cat*_ values than T0099 and SC at 25° and 45°C (Table 1); these results, based on AAPF hydrolysis, are consistent with the results from the casein digestion assay (Figure 7) where P355 and T0034 demonstrated a higher activity. T0034 had the highest *K*_*m*_ at 25° and 45°C, closely followed by P355, whereas *K*_*m*_ of SC and T0099 are lower. *K*_*m*_ increased between 0.02 and 0.06 mM with higher temperatures for every protease, except for T0034, which decreased from 1.83 to 1.26 mM. SC displayed the highest catalytic efficiency (*k*_*cat*_/*K*_*m*_) at 25°C of all proteases, while P355 and T0034 displayed the highest at 45°C. The catalytic efficiency increased approximately 1.5 to 2.5-fold for every protease from 25 to 45°C, except for T0034, which quadrupled from 2.09 × 10^5^ to 8.55 × 10^5^ M^–1^ s^–1^. The kinetic data (Table 1) indicate that P355 and T0034 are cold-adapted ISPs due to higher *k*_*cat*_ and *K*_*m*_ values at both temperatures tested relative to the reference enzymes T0099 and SC. However, T0099 and SC showed similar catalytic efficiency to P355 and T0034 owing to their low *K*_*m*_ values.

**TABLE 1:** Derived kinetic constants.

Protease	T (°C)	*K*_*m*_ (mM)	*k*_*cat*_ (s^–1^)	*k*_*cat*_/*K*_*m*_ (M^–1^ s^–1^)
P355	25	1.12 ± 0.02	386 ± 3	3.45 × 10^5^
	45	1.17 ± 0.02	996 ± 9	8.52 × 10^5^
T0034	25	1.83 ± 0.05	382 ± 5	2.09 × 10^5^
	45	1.26 ± 0.03	1077 ± 11	8.55 × 10^5^
T0099	25	0.10 ± 0.01	35 ± 1	3.49 × 10^5^
	45	0.12 ± 0.01	69 ± 1	5.73 × 10^5^
SC	25	0.38 ± 0.01	208 ± 1	5.48 × 10^5^
	45	0.44 ± 0.01	348 ± 2	7.91 × 10^5^

Deviations show the confidence interval (0.99), *n* = 3.

### Reduced and alkylated BSA is readily digested by P355 and T0034 at around 0°C

**FIGURE 8:**
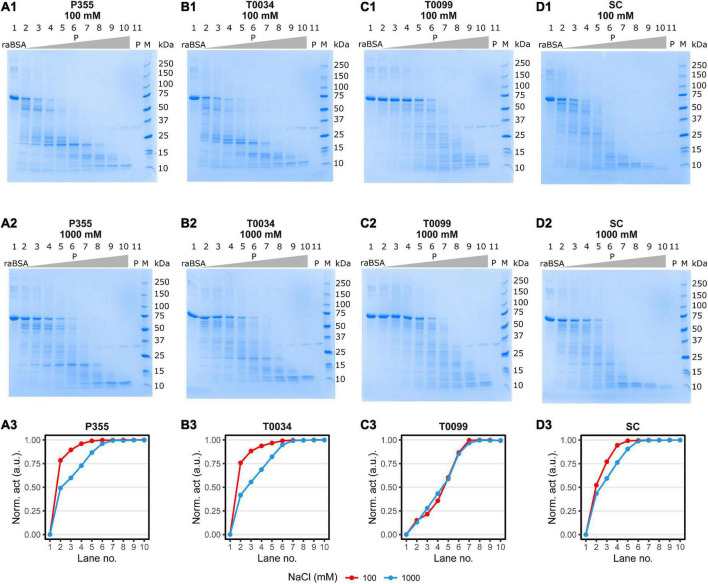
raBSA digest in ice-water (0°C). 3.75 μg (1.88 μM) raBSA (approximately apparent 70 MW) was digested by an increasing concentration of matured protease for 2 h in ice-water in the presence of 100 or 1000 M NaCl before being heated to 95°C for 10 min. The protease concentration in each lane was as follows: (1) 0nM, (2) 0.4 nM, (3) 0.8 nM, (4) 1.6 nM, (5) 3.1 nM, (6) 6.3 nM, (7) 12.5 nM, (8) 25 nM, (9) 50 nM, (10) 100 nM, and (11) 100 nM. Lane 11 contained no raBSA. The digestion patterns of P355, T0034, T0099, and Subtilisin Carlsberg (SC) and the corresponding degree of raBSA digestion are shown in panels **(A1–3,B1–3,C1–3,D1–3)**, respectively. The normalized activity values were obtained by normalizing the intact raBSA bands (lane 2 through 10) to the intact raBSA band control (lane 1) and subsequently subtracted from 1. Hence, the activity values correspond to a relative reduction in band intensity. P355 and T0034 were most efficient in digestion raBSA: They lowered the intensity of the BSA band of approx. 75% at 0.4 nM enzyme, while T0099 and SC had lowered the intensity with approx. 20 and 50%, respectively. The increase to 1000 mM NaCl decreased the ability of all proteases, most prominent for P355 and T0034, although P355 was slightly more efficient in digestion raBSA compared to T0034 at 1000 mM. SC was slightly inhibited by 1000 mM NaCl, while T0099 was largely unaffected. raBSA, reduced and alkylated BSA; P, protease.

A critical trait in cold-adaptation is higher catalytic activity at low temperatures compared to meso- and thermophilic conditions (Åqvist et al., 2017). We tested the proteolysis at low temperature by incubating an equal molar titration series of P355, T0034, T0099, or SC with a fixed amount of reduced and alkylated BSA (raBSA) at 0°C (ice water). Digestion of the raBSA substrate was tested at low (0.1 M) or high (1 M) concentration of NaCl salt to examine whether the same effect was observed for raBSA as with casein. The digestion of raBSA were in line with the kinetic data (Table 1) and casein digestion (Figure 7B); P355 and T0034 degraded raBSA quicker than T0099 and SC. It was observed that 1 M NaCl inhibited proteolytic activity for every protease, except for T0099, where activity was largely unaffected by NaCl at all protease concentrations (Figure 8) in line with the casein digest (Figure 7). P355 (Figure 8, panels A1–3) and T0034 (Figure 8, panels B1–3) followed the same pattern: at the highest substrate:protease ratios, the semi-quantitative band intensities of intact raBSA was reduced by >75% in 0.1 M NaCl. Increasing the NaCl concentration to 1 M resulted in less efficient hydrolysis by P355, T0034 and SC, i.e., cleaving about 40–50% raBSA. T0099 was the least efficient enzyme in this assay, showing a reduced band intensity by around 20% at the same substrate:protease ratios at both NaCl conditions (Figure 8, panels C1–3). SC showed intermediate digestion capabilities: at the highest substrate:protease rations conditions, SC reduced the intact raBSA band intensity with approximately 50% in 0.1 M NaCl, with a slight inhibition by higher NaCl concentration (Figure 8, panels D1–3). These results align with the casein digestion assay (Figure 7B). The position of the degradation bands from raBSA were not identical between the proteases, indicating different specificities. We employed mass spectrometry (MS) to investigate digestion products to examine whether there were differences in hydrolysates of raBSA depending on the amino acid at position P1. MS/MS analysis of peptide products revealed that the proteases cleaved at the same residues, which included hydrophobic, basic, and acidic residues (see Section “Data availability statement”). Additionally, NaCl also changed the degradation pattern slightly. For instance, at the 25 kDa marker in Figure 8, panels A1–2, a few bands disappear, which indicate changes in substrate specificity. However, this was not investigated further.

## Discussion

### P355, T0034, and T0099 depend on calcium for maturation and activity

The goal of this paper was to identify, produce, and characterize a novel protease from the cold-adapted and halo-tolerant bacterium *P. halocryophilus* Or1, which can grow down to −15°C (Mykytczuk et al., 2013). We identified a gene encoding a putative intracellular subtilisin protease (ISP) denoted P355 in the genome sequence, which we expected to harbor cold-adapted traits. We successfully cloned and produced the recombinant P355 protease in *E. coli* and characterized the enzyme. In addition, we compared it to homologous ISPs and an ESP identified from literature or databases: The putative mesophilic T0034 from *Planococcus* sp. AW02J18 (Bjerga et al., 2018), the presumably thermostable T0099 from *Parageobacillus thermoglucosidasius* and the ESP industry reference Subtilisin Carlsberg (SC). The results are summarized in Table 2.

**TABLE 2:** Collected and summarized results obtained in this study.

		P355	T0034	T0099	SC	Figure/Table
Subtilisin type		ISP	ISP	ISP	ESP	Supplementary Figure 2
Maturation dependent on calcium		Yes	Yes	Yes	Not examined	Figure 1
Maturation time course (min)		90	120	60	Not examined	Figure 1
Activity dependent on calcium		Yes	Yes	Yes	No	Supplementary Figure S3
Inhibition by EDTA		Yes	Yes	Yes	No	Supplementary Figure 10A
Inhibition by Pefabloc^®^		Yes	Yes	Yes	Yes	Supplementary Figure 10B
pH activity		Alkaline	Alkaline	Alkaline	Alkaline	Figure 2
pH stability range		5.5–10.5	6.5–10.5	4.5–10.5	4.5–10.5	Figure 3
Temperature optimum (°C)		50	60	50	50	Figure 4
Temperature stability		Lowest	Low	Highest	High	Figure 5
*k*_*cat*_ (s^–1^)	45°C 25°C	386 ± 3	382 ± 5	35 ± 1	208 ± 1	Table 1
		996 ± 9	1077 ± 11	69 ± 1	348 ± 2	
Relative casein/raBSA digest efficiency		High	High	Low	Intermediate	Figures 7B, 8
Activity affected by increasing [NaCl]	AAPF	Increased	Increased	Small effect	Small effect	Figure 6C
	Casein/raBSA	Decreased	Decreased	Decreased	Decreased	Figures 7A, 8
Cleavage sites observed		Hydrophobic, acidic, and basic	Hydrophobic, acidic, and basic	Hydrophobic, acidic, and basic	Hydrophobic, acidic, and basic	See section “Data availability statement”

The ISPs, P355, T0034, and T0099, showed a dependency on calcium for activation (Figure 1 and Supplementary Figure 3), and up to 2 h were required for complete processing in order to gain full activation (Figure 1). N-terminal sequencing confirmed the removal of the pro-peptide and its LIPY/F motif in every ISP. We also observed maturation intermediates for P355 and T0099, indicating that activation may be a multi-step process. No intermediates were observed for T0034, in contrast to what has been previously reported (Bjerga et al., 2018). However, maturation is calcium- and pH-dependent (Bjerga et al., 2018) and experimental variation in assay conditions may explain the deviation to the previous report. In this study, the combination of a higher calcium concentration and higher pH compared to the previous study on T0034 (Bjerga et al., 2018) likely caused a swifter maturation and lack of observable intermediates. Inhibition of the ISPs by EDTA (Supplementary Figure 10A) shows that not only is calcium necessary for ISP activation, but it is also needed to sustain activity in the ISPs in this study. In contrast, the ESP SC was largely unaffected by the presence of EDTA.

Gamble et al. (2011) reported a lag phase during the maturation of an ISP from *Bacillus clausii* NN010181 before an exponential increase in active ISP followed by a plateau. This is due to a positive feedback mechanism where protease removes the N-terminal pro-peptide and activates the unprocessed protease (Gamble et al., 2011). Lag phases were not observed in this study, possibly due to a more rapid maturation at higher pH values and calcium concentrations.

### The matured proteases are active and stable in alkaline pH

Alkaline activity is expected for serine proteases, as the His and Asp residues of the catalytic triad must be in their deprotonated form to deprotonate Ser (Hofer et al., 2020). Every protease in this study showed activity and stability in the alkaline range.

T0034 has been characterized previously by Bjerga et al. (2018), and we chose to include this close homolog in comparative studies of P355 due to their relatively high sequence homology. This and the past study of T0034 report some differences in pH profiles. We both find maximum activity at a plateau around pH 9–11, but we also found that T0034 maintain activity at both pH 6.5 and 7.0 (Figure 2B), whereas Bjerga et al. found little to no activity at pH 7 and lower. We could reproduce the results from Bjerga et al. when calcium was added immediately prior to performing the assays (data not shown), allowing a pH-dependent progression of maturation taking place during the assay. Experimental data included in this study are from assays where the enzymes were pre-incubated 2 h with calcium to ensure a complete maturation. We therefore propose that these differences can be explained by experimental variations. For activity assessment, we thus recommend that complete maturation should be achieved prior to assaying.

### NaCl affects the activity depending on substrate size

Increasing NaCl and KCl concentrations enhanced P355 and T0034 activity when the peptide AAPF was used as a substrate, while T0099 and SC were largely unresponsive in comparison (Figures 6C, D). However, increasing concentration of NaCl decreased activity for all proteases when the larger substrates casein and raBSA were used (Figures 7B, **8**, panels A3, B3, and D3), apart from T0099 activity against raBSA, which was not affected by NaCl (Figure 8, panel C3). NaCl can favor a particular structural conformation and indirectly influence catalytic activity (Ortega et al., 2011). NaCl and KCl could favor a more rigid structural conformation for P355 and T0034 where a small substrate, e.g., the AAPF peptide, can easily enter the catalytic cleft. The same putative rigid structure could at the same time make it more difficult for a larger substrate to fit into the catalytic cleft. Mykytczuk et al. (2013) report a high copy number of osmolyte transporters in *P. halocryophilus* Or1 could indicate a high intracellular ionic strength. Whether this is also the case for *Planococcus* sp. AW02J18 is uncertain as the genome sequence is unknown.

### P355 has cold-adaptive traits

We conducted an array of experiments to determine whether P355 is, in fact, a cold-adapted protease, including temperature optimum and stability, salt stability, Michaelis-Menten kinetics at two temperatures, and digestion of a large substrate at 0°C.

Thermal stability and temperature optimum are usually reduced for cold-adapted enzymes compared to their meso- and thermophilic counterparts (Siddiqui and Cavicchioli, 2006; Gerday, 2014). The proteases in this study showed increasing stability against thermal inactivation in the following order: P355, T0034, SC, and T0099 (Figure 5). This showed that P355 is the most heat-labile protease in this study. Ice water (0°C) preserved the activity of the proteases after 120 h of incubation. Strongin et al. (1978) reported an ISP from *B. subtilis*, which retained all its activity after 2 months at 4°C. One should keep in mind that *in vivo* and *in vitro* experiments can differ; molecular crowding, molecular interactions, chaperones, and translation rates can influence protein stability (McGuffee and Elcock, 2010; Gershenson and Gierasch, 2011). Simply adding BSA has shown to enhance protease stability (Narinx et al., 1997).

The temperature optimum of P355 is equal to that of the putative thermophile T0099 (50°C), in contrast to what might be expected from a cold-adapted protease. However, it is not unheard of that some cold-active enzymes display a relatively high-temperature optimum (Siddiqui and Cavicchioli, 2006). The temperature optimum of T0034 determined in this study (60°C) is higher than previously determined by Bjerga et al. (45°C) (Bjerga et al., 2018). This is most likely due to the difference in experimental approaches with and without a calcium-maturation step before assaying as described for the pH optimum experiments. The explanation for the higher temperature optimum of T0034 in this study is likely due to an improved thermal stability in the presence of calcium (Voordouw et al., 1976; Veltman et al., 1998; Bjerga et al., 2018). P355 showed much less activity at 65°C than the others due to its instability at 65°C (Figure 5).

Chemical denaturation is usually correlated with thermal lability (D’Amico et al., 2003). This trend is observed for urea (Figure 6A), where increasing concentrations inactivate the ISPs and the ESP, and where P355 and T0034 are somewhat more sensitive to urea than the two other enzymes. Denaturation with Gnd (Figure 6B) exhibits a more complex pattern with overlapping graphs: at lower range concentrations P355 activity is slightly enhanced, peaking at 125 mM Gnd, but a Student’s *t*-test indicated no significant difference (*p* < 0.05) between the lowest Gnd concentration (3.9 mM) and 125 mM Gnd. Overall, SC is the most stable protease in Gnd, while ISPs, and in particular T0034, are less stable.

Other indications of cold adaption in P355 are its relatively high catalytic constant (*k*_*cat*_) and *K*_*m*_ compared to SC and T0099 (Table 1). The general increase in *k*_*cat*_ from 25 to 45°C likely accounts for the increase in *K*_*m*_. However, *K*_*m*_ for T0034 decreased, which explains why the catalytic efficiency increased more for T0034 compared to the other proteases. The order of thermal and chemical (urea) stability correlates with *k*_*cat*_, i.e., P355 has traded stability for higher activity (Santiago et al., 2016). A higher *k*_*cat*_ is explained by a lower energy of activation due to fewer intramolecular bonds–accompanied by a higher entropy of activation–that needs to be broken during a chemical reaction (Feller and Gerday, 2003; Siddiqui and Cavicchioli, 2006). SC showed intermediate values in line with previously reported parameters (Evans et al., 2000). *k*_*cat*_ for cold-adapted proteases decreases at a lower rate compared to thermophilic counterparts with decreasing temperature (Åqvist et al., 2017). However, this was not the case for neither P355 nor T0034; their *k*_*cat*_ decreased relatively fast from 45 down to 25°C compared to T0099 and SC. Even so, *k*_*cat*_ for P355 and T0034 were still higher than T0099 and SC at both temperatures in line with the casein (Figure 7B) and raBSA digest assays (Figure 8). The digestion of raBSA demonstrates that P355 and T0034 have relatively high activity at 0°C compared to T0099 and SC. The tendencies discussed above regarding the kinetic assay and the casein and raBSA digestion assays still hold true when taking the relatively lower number of active sites of T0099 into account, which was roughly half compared to P355, T0034, and SC (Supplementary Figures 6–9). *k*_*cat*_ of T0099 and digestion of casein and raBSA would approximately double but T0099 would still exhibit the lowest activity of all 4 proteases. The degradation patterns of raBSA differed, which likely reflects differences in substrate specificities between the proteases. MS/MS analysis of digested raBSA indicated a broad substrate specificity of the proteases used in this study; all proteases were able to cleave at hydrophobic, basic, and acidic residues at the P1 location. This has previously been shown for SC (Evans et al., 2000). They were also capable of cleaving at modified Cys residues (alkylated through reaction with iodoacetamide for MS analysis). An ISP able to cleave after Lys and hydrophobic residues has been reported previously (Gamble et al., 2012).

In summary, we produced and characterized a novel cold-adapted ISP (P355) from the *P. halocryophilus* Or1 genome. The putative mesophilic T0034 turned out to be a cold-adapted ISP like P355 and both showed activity-stability trade-off compared to T0099 and SC. All proteases in this study were active and stable at alkaline pH. P355 showed the highest thermal and chemical lability followed closely by T0034. Titration with NaCl and KCl enhanced P355 and T0034 activity considerably when using AAPF as a substrate, while NaCl decreased activity when using casein or raBSA as substrate, suggesting that substrate binding is affected by the ionic strength of the buffer. The *k*_*cat*_ values correlated well with the degree of casein and raBSA digestion; both P355 and T0034 exhibited higher *k*_*cat*_ values compared to T0099 and SC, which likely accounts for the higher digestion efficiency of casein (RT) and raBSA digestion (0°C) in line with the activity-stability trade-off characteristic in cold adaptation. Altogether, we conclude that P355 along with T0034 are cold-adapted proteases.

## Data availability statement

The datasets presented in this study can be found in online repositories. The names of the repository/repositories and accession number(s) can be found in the article/Supplementary material.

## Author contributions

CR and JE: conceptualization. CR and SH: methodology. CR: software, formal analysis, data curation, writing—original draft, and visualization. CR and IT: investigation. JE, ØL, and GB: resources. CR, MT, SH, ØL, GB, PS, and CS: writing—review and editing. JE and MT: supervision and project administration. JE and CS: funding acquisition. All authors contributed to the article and approved the submitted version.
