# Supplementary material for: Characterization of a novel cold-adapted intracellular serine protease from the extremophile Planococcus halocryophilus Or1
Source: Front Microbiol. 2023 Feb 23;14:1121857. doi: 10.3389/fmicb.2023.1121857 (PMC9995970; doi:10.3389/fmicb.2023.1121857)
Supplement: Supplementary file 1 [file Data_Sheet_1.docx]

**Supplementary Material**

**Characterization of a novel cold-active intracellular serine protease from the extremophile *Planococcus halocryophilus* Or1**

Casper Bøjer Rasmussen^1^, Carsten Scavenius^2^, Ida B. Thøgersen^1^, Seandean Lykke Harwood^1^, Øivind Larsen^3^, Gro Elin Kjæreng Bjerga^3^, Peter Stougaard^4^, Jan J. Enghild^1^, Mariane Schmidt Thøgersen^4^*

^1^Department of Molecular Biology and Genetics, Aarhus University, Universitetsbyen 81, 8000 Aarhus C, Denmark

^2^Danish Technological Institute, Kongsvang Alle 29, 8000 Aarhus C

^3^NORCE Norwegian Research Centre AS, P.O. Box 22 Nygårdstangen, 5838 Bergen, Norway

^4^Department of Environmental Science, Aarhus University, Frederiksborgvej 399, 4000 Roskilde, Denmark

*Corresponding author: [mst@envs.au.dk](mailto:mst@envs.au.dk)

**Supporting information in this document**

[Materials and Methods 3](#_Toc126952953)

[Pore limited native PAGE 3](#_Toc126952954)

[Protease titration against α-2-macroglobulin 3](#_Toc126952955)

[Supplementary information to supplementary figure S5-9 3](#_Toc126952956)

[Using α2M to evaluate the number of active sites 3](#_Toc126952957)

[Supplementary figures and tables 5](#_Toc126952958)

[Figure S1. SDS-PAGE of purified proteases from different batches. 5](#_Toc126952959)

[Figure S2. Amino acid alignment. 6](#_Toc126952960)

[Figure S3. Calcium titration. 7](#_Toc126952961)

[Figure S4. Entire SDS-PAGE of figure 1. 8](#_Toc126952962)

[Figure S5: Trypsin titration against α2M. 9](#_Toc126952963)

[Figure S6. P355 titration against α2M. 10](#_Toc126952964)

[Figure S7. T0034 titration against α2M. 11](#_Toc126952965)

[Figure S8. T0099 titration against α2M. 12](#_Toc126952966)

[Figure S9. SC titration against α2M. 13](#_Toc126952967)

[Figure S10. Inhibition with EDTA and Pefabloc®. 14](#_Toc126952968)

[Figure S11. Michaelis-Menten kinetics at 25 and 45°C of matured proteases. 15](#_Toc126952969)

[Figure S12. Residual plots from Michaelis-Menten fitting. 16](#_Toc126952970)

[Table S1. Statistics from the Michaelis-Menten fitting. 17](#_Toc126952971)

[Data availability 18](#_Toc126952972)

[References 18](#_Toc126952973)

# Materials and Methods

## Pore limited native PAGE

Samples were mixed 1:1 with native PAGE buffer containing 20% (V/V) and bromphenol blue. Proteins were separated in 5-10% acrylamide gradient gels casted in-house (10 × 10 × 0.15 cm) in TBE buffer (89 mM Tris, 89 mM boric acid, and 2 mM EDTA) (Manwell, 1977). The voltage was set to 100 volts with a dynamic ampere. The gels were developed with Coomassie Brilliant Blue. Imaging and densitometry were done in a Gel Doc™ EZ imager (Biorad).

## Protease titration against α-2-macroglobulin

The ISPs (P355, T0034, and T0099) and the ESP (SC) were matured for 2 h as previous described, however, glycine concentration was 10 mM. α-2-macroglobulin (α2M) was purified as described in (Harwood et al., 2022). The subtilisins along with trypsin were incubated with α2M at different molar ratios ranging from 0-2.91 mol/mol of protease:α2M in 80 mM HEPES, 100 mL NaCl, and 5 mM CaCl_2_, pH 7.4, for 1 h at 37°C. α2M was also treated with 250 mM methylamine (pH 8) for 2 h at 37°C in 250 mM HEPES, 100 mM NaCl, 5 mM CaCl_2_, pH 7.4 ) to visualize the ‘fast’ band. See supplementary text for further description of α2M.

# Supplementary information to supplementary figure S5-9

## Using α2M to evaluate the number of active sites

We attempted to active site titrate the proteases to determine the percentage of active protease in the activated protease stocks used for characterization experiments by using p-guanidinobenzoate hydrochloride (pNPGB) (Chase and Shaw, 1967; 1969) and N-trans-cinnamoylimidazole (Schonbaum et al., 1961) by measuring remaining activity. Both compounds react with the catalytic triad serine of serine proteases. However, the acyl-P355, -T0034, -T0099, and -SC were unstable in pH range (data not shown) where the proteases were catalytically active (Fig. 2 and Fig. 3). The instability of guanidinobenzoyl-protease was visible as a nonlinear increase in absorbance at 405 nm (data not shown), which reflected the continual digestion of substrate even after saturating inhibitor concentrations. Differences in acyl-enzyme stability has been reported previously: Chase and Shaw showed complete diacylation of guanidinobenzoyl-trypsin after 50 h and guanidinobenzoyl-thrombin (human and bovine) after 1.5 h (Chase and Shaw, 1969). Furthermore, the burst-phase was too quickly to detect even at low pH values (data not shown).

As an alternative to active site titration with small molecule inhibitors, we used α-2-macroglobulin (α2M) to roughly evaluate the relative concentrations of active protease in our stocks. α2M is a 720 kDa protease inhibitor with a unique trapping mechanism. Proteases from every class can hydrolyze a peptide bond in the bait region of α2M (Gettins and Cunningham, 1986; Sottrup-Jensen et al., 1989), which then collapses around the proteases thus hindering the protease’s hydrolysis of additional protein substrates (Barrett et al., 1979). Upon collapsing, α2M changes from an electrophoretically ‘slow’ form to a ‘fast’ form on a pore-limited PAGE (Barrett et al., 1979). Importantly, α2M reacts with a specific stoichiometry with proteases, forming up to a ≈2:1 molar ratio protease:α2M complexes with most small proteases such as trypsin; however, at a 1:1 ratio all α2M is converted to its ‘fast’ form if the protease is added at once (Larsson et al., 1989). The decrease in band density of the ‘slow’ band on a pore-limited PAGE with an increasing protease concentration was used to approximate the relative number of active sites. This was done by performing densitometry on the ‘slow’ bands and plotting against the protease:α2M ratio (Fig. S5-9). Linear regression was performed on the data points indicated by a solid bar on the gel where non-saturating amounts of protease had been added. For simplicity, only the data points in this range where the responses were most linear are shown in the plots. Trypsin forms a stable acyl-complex (Chase and Shaw, 1967; 1969) and was successfully active site titrated with pNPGB. Thus, active site titrated trypsin with a known molar concentration of active protease was also titrated against α2M to serve as a positive control (Fig. S5). Although a comparison of the other slopes with that of trypsin could in principle be used to calculate an absolute percentage of active protease in our stocks, this assumes complete mechanistic equivalence in the reaction of all these proteases by α2M and would risk over-interpreting the data. Instead, we conclude from these slopes that trypsin, P355, T0034, and SC contain roughly the same number of active sites, while T0099 contain approximately half as many.

# Supplementary figures and tables


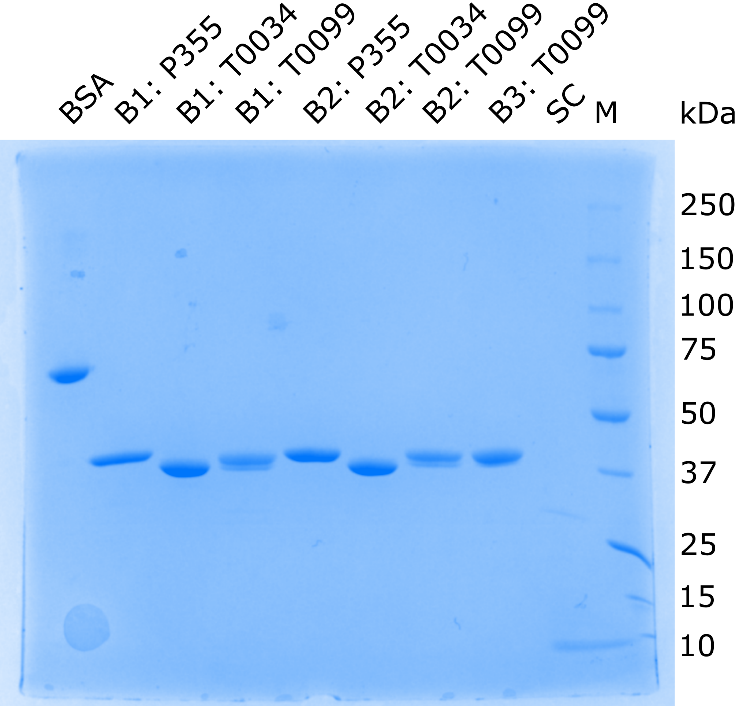


Figure S1. SDS-PAGE of purified proteases from different batches. 2 µg of each protease was loaded, except SC where 1.6 µg was loaded. The faint band of SC is due to a lower substance amount loaded, but mostly due to poor Coomassie affinity partly explained by a low content of Arg residues, which Coomassie binds to. B1 = batch no. 1, B2 = batch no. 2, B3 = batch no. 3, M = marker.


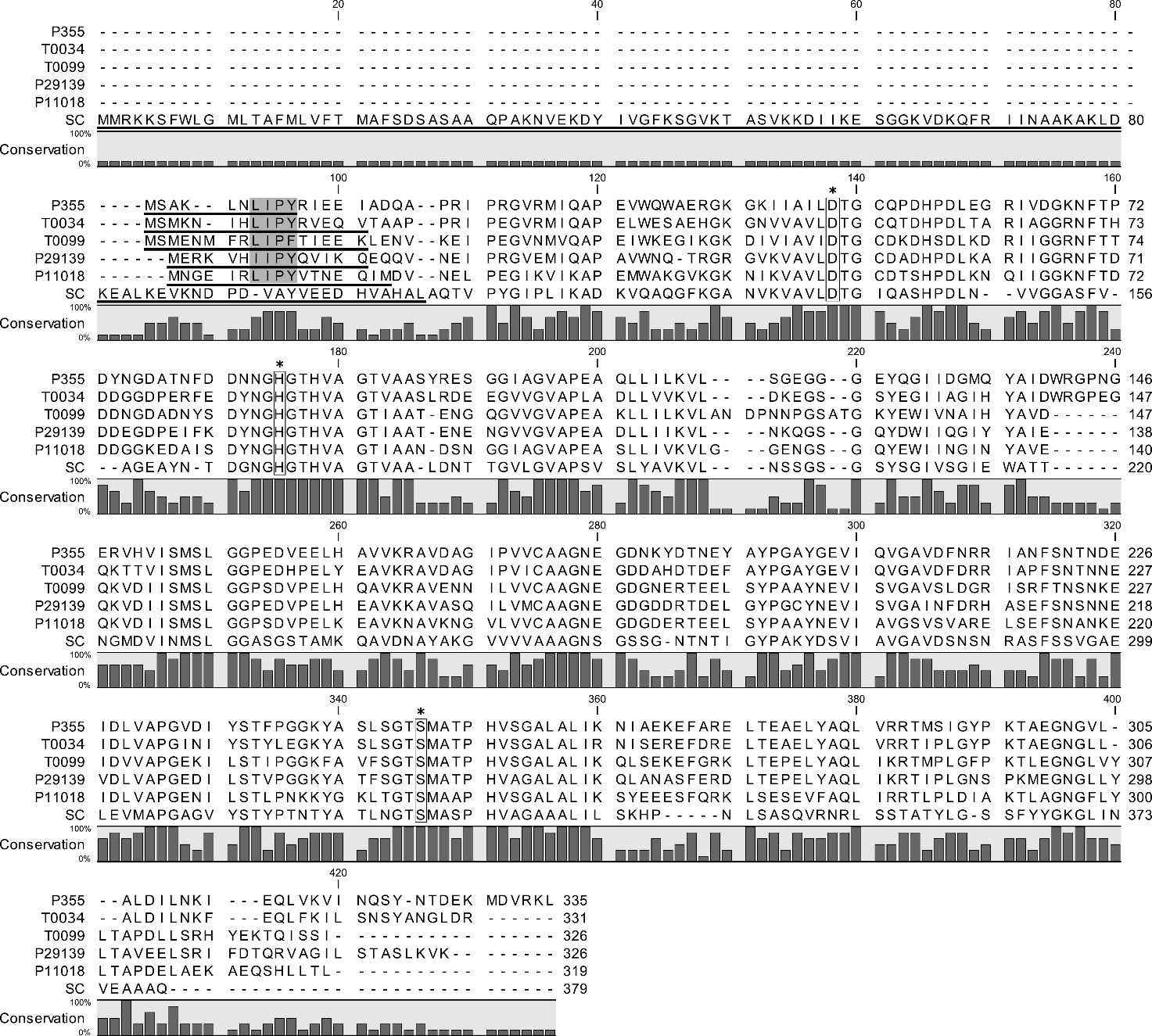


Figure S2. Amino acid alignment. Note that the N-terminal Met is included – despite being removed during translation/purification – and the affinity-tag (AHHHHHH) was excluded for the ISPs P355, T0034, and T0099 in the alignment. Alignment was done in CLC Main Workbench (version 22.0). The bars show the degree of conservation across all 4 proteases. Underlined sequences indicate the pro-peptide that is removed during maturation. The L/IIPY/F motif is highlighted with a grey box in the ISPs and the conserved residues involved in the catalytic triad (D, H, and S, alignment sequence position 138, 175, and 346, respectively) are marked with asterisks and framed. Residues are conserved around the residues involved in the catalytic triad. Note that T0034 contains two additional residues in the N-terminal – Met and Ser – in the N-terminus compared to the previously reported sequence (40).


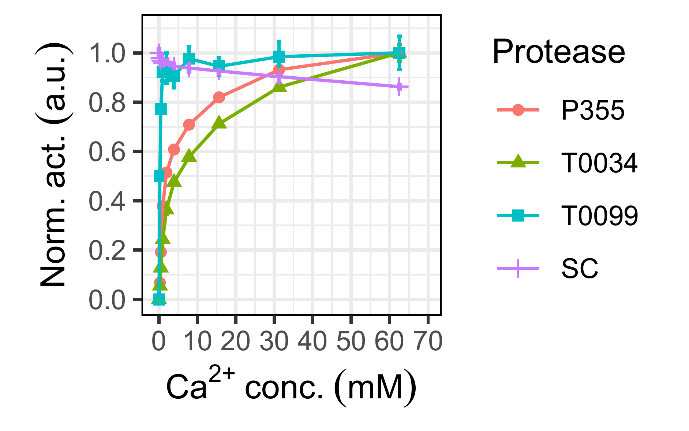


Figure S3. Calcium titration. The proteases were incubated in 100 mM NaCl, 100 mM glycine pH 9.5, and 0.1% Triton X-100 and varying CaCl_2_ ranging from 0-62.5 mM for 30 min before activity was measured with 0.2 M AAPF. Activity was normalized to the highest activity for the given protease. P355, T0034, and T0099 are dependent on CaCl_2_ for activity, while SC activity decreased albeit to a low degree. Error bars show standard deviation (n = 3). Note that some error bars are within the marker.


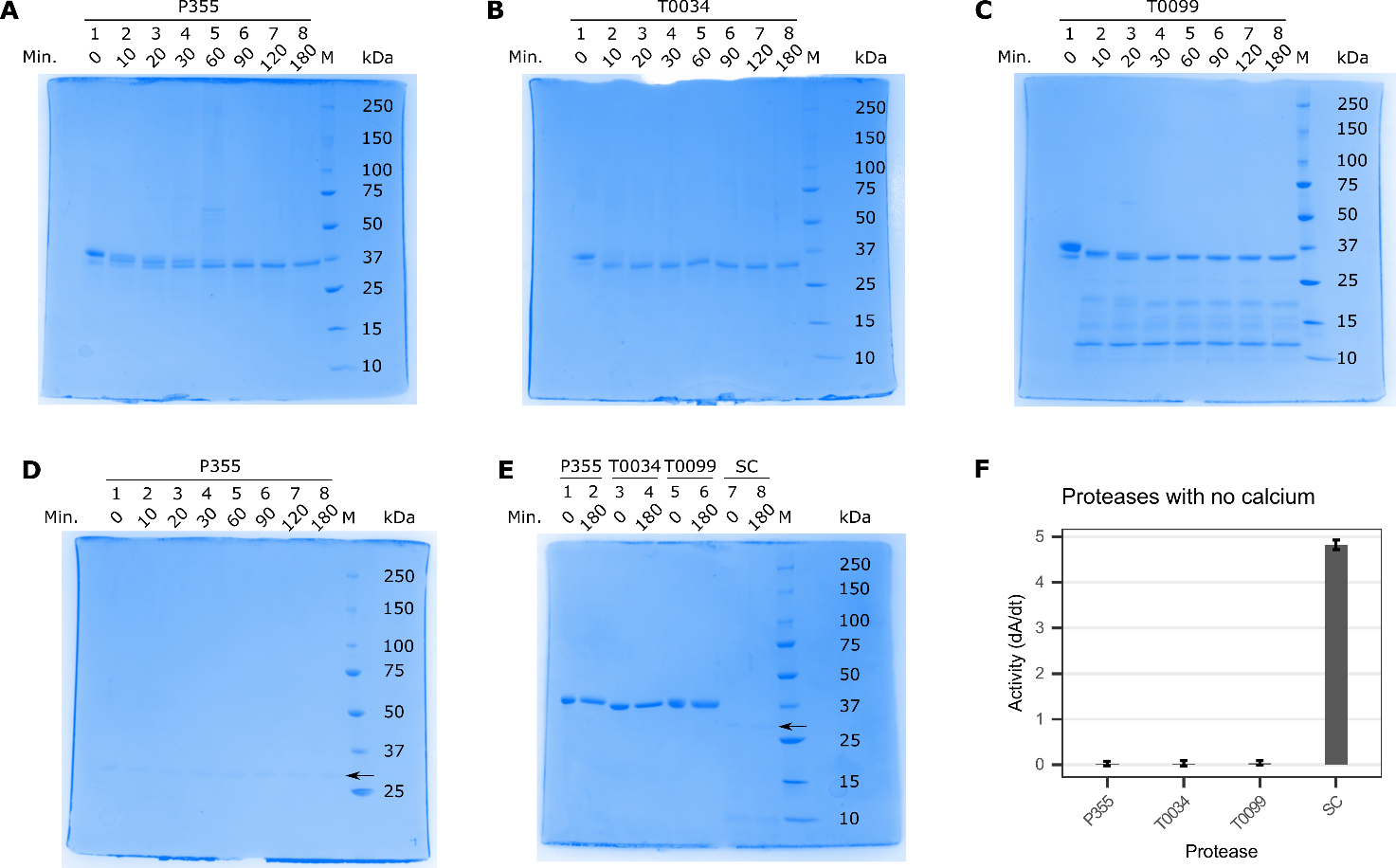


Figure S4. Entire SDS-PAGE of figure 1. **A)** P355. A higher molecular band can be seen in lane 5. This is an experimental artifact. **B**) T0034, respectively. **C**) The entire image of T0099 reveals additional bands, which is not seen for the other proteases. The intensities of these lower molecular weight bands do not change over time. **D)** SC is seen as faint band at 27.3 kDa (arrow). **E)** Incubation of proteases without 25 mM calcium for 0 and 180 min. Arrow indicates SC. **F)** Protease activity without calcium after 180 min incubation without calcium. M = marker.


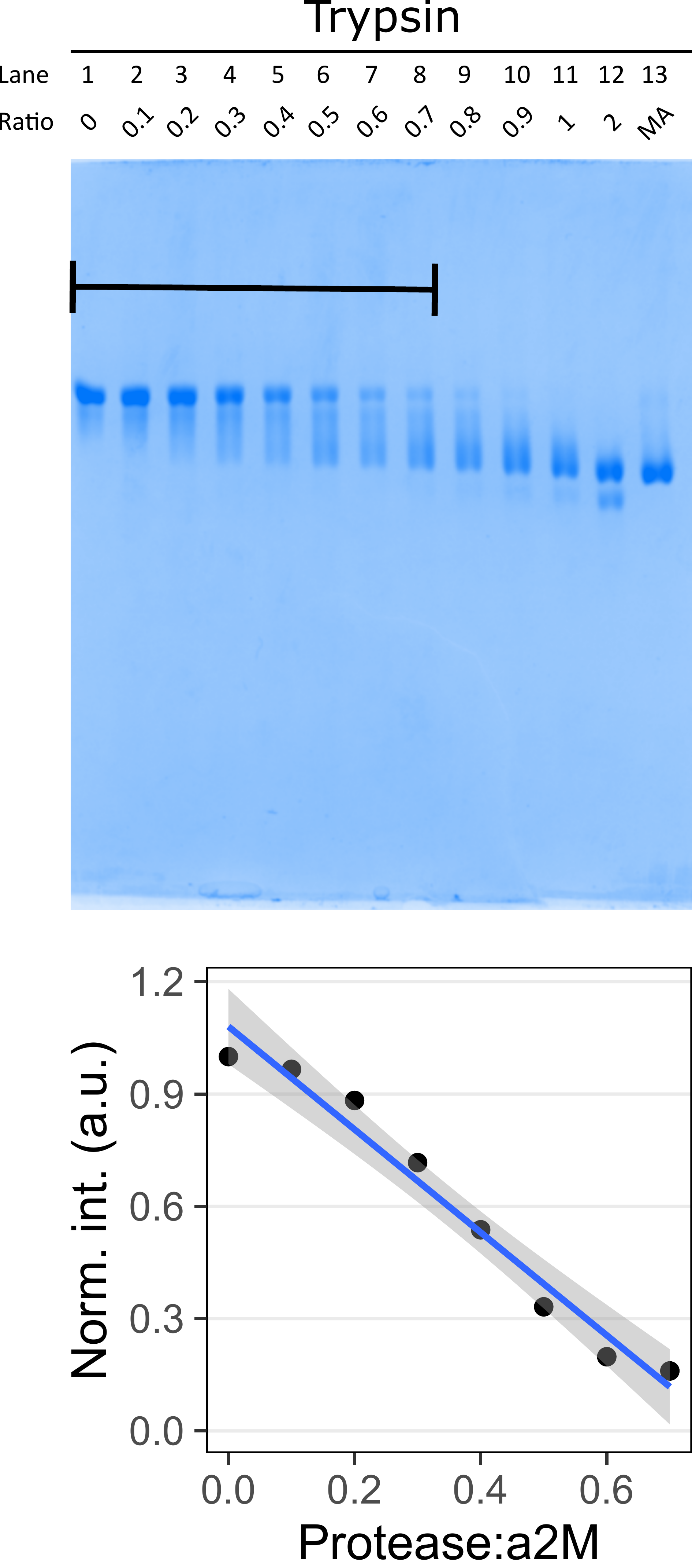


Figure S5: Trypsin titration against α2M. Proteases were titrated against α2M. Ratios indicate molar stoichiometry between protease and α2M. The molarity of trypsin is based on the active site concentration. The band intensity was normalized to the band with no added protease (lane 1) for the given protease. The solid bars on the gels indicate the range used for linear regression (blue line) with the derived linear equation shown in the plot. The grey area indicates the confidence interval (0.95) of the fit.


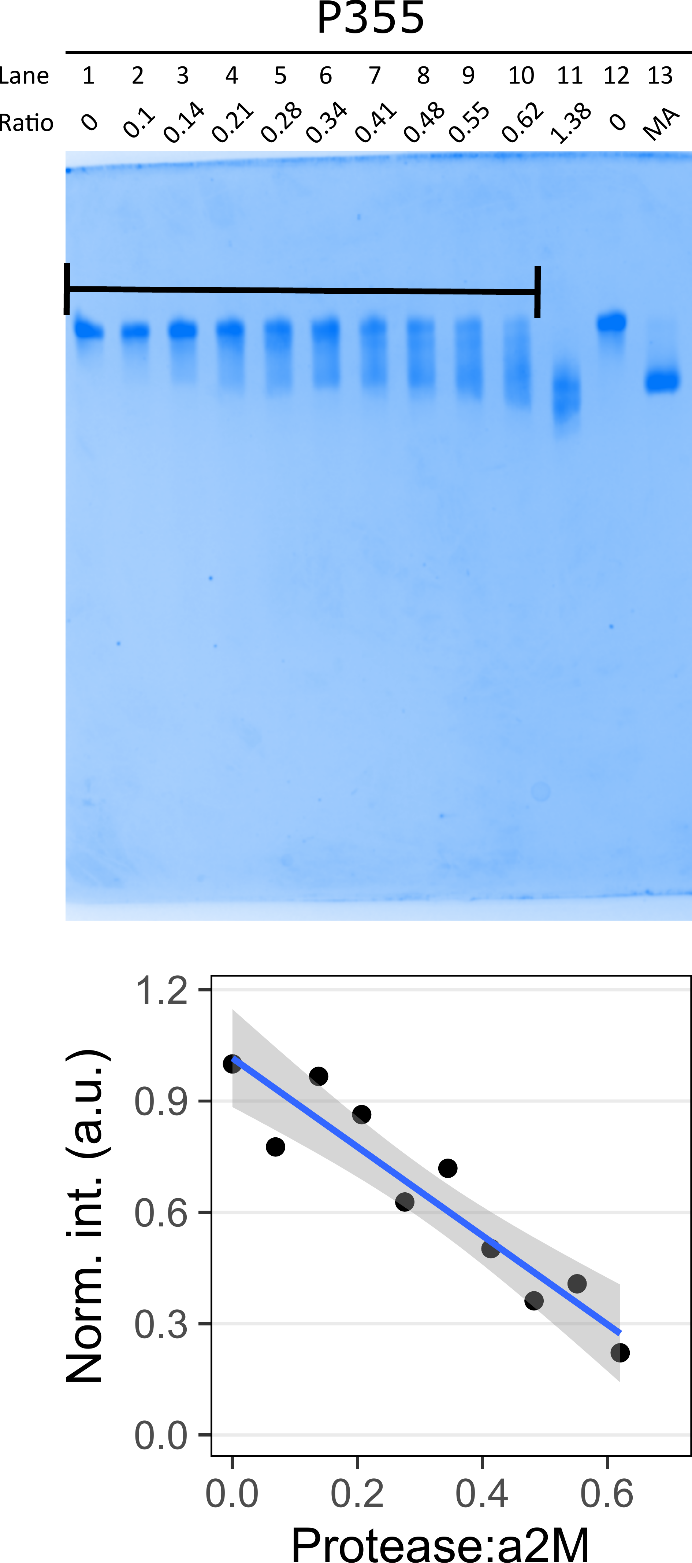


Figure S6. P355 titration against α2M. Ratios indicate molar stoichiometry between protease and α2M. The molarity of P355 is based on the total protease concentration. The intensity was normalized to the band with no added protease (lane 1) for the given protease. The solid bars on the gels indicate the range used for linear regression (blue line) with the derived linear equation shown in the plot. The grey area indicates the confidence interval (0.95) of the fit.


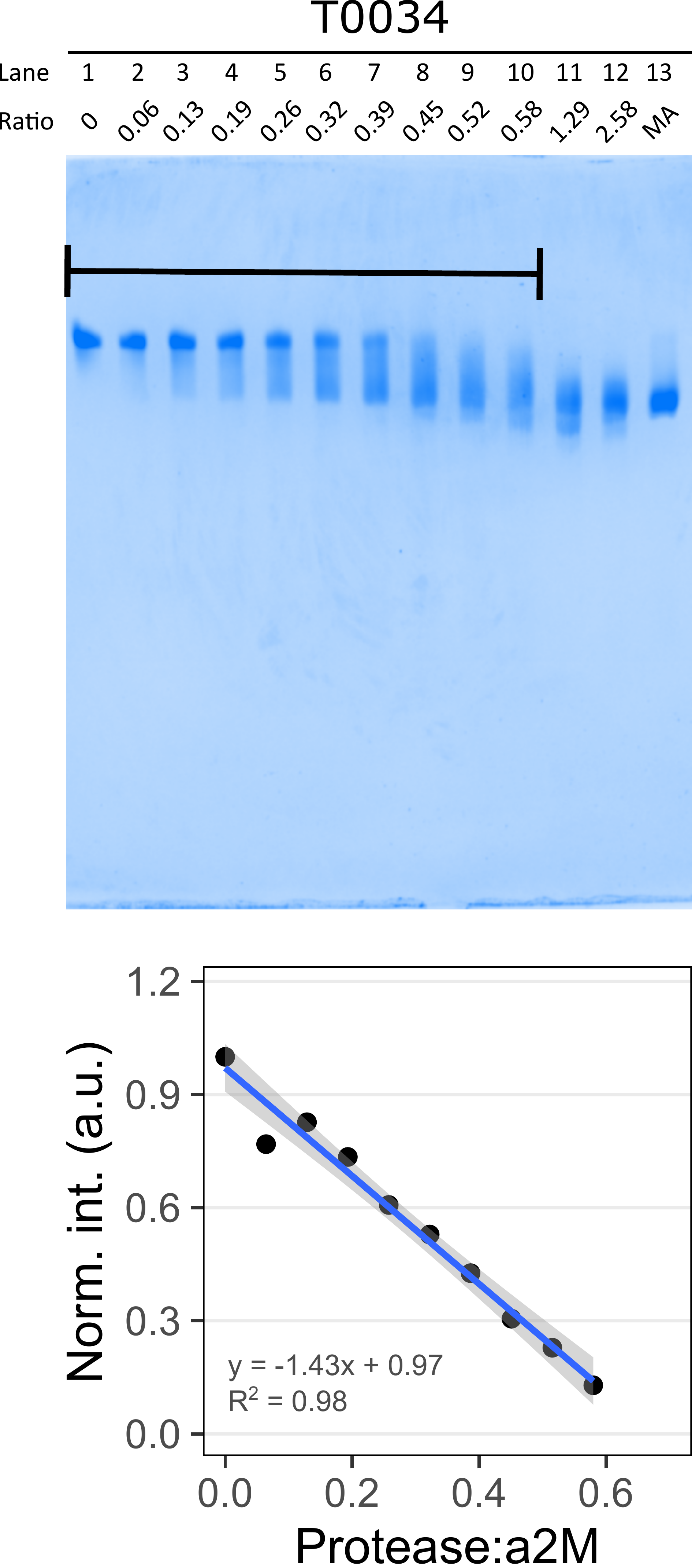


Figure S7. T0034 titration against α2M. Ratios indicate molar stoichiometry between protease and α2M. The molarity of P355 is based on the total protease concentration. The intensity was normalized to the band with no added protease (lane 1) for the given protease. The solid bars on the gels indicate the range used for linear regression (blue line) with the derived linear equation shown in the plot. The grey area indicates the confidence interval (0.95) of the fit.


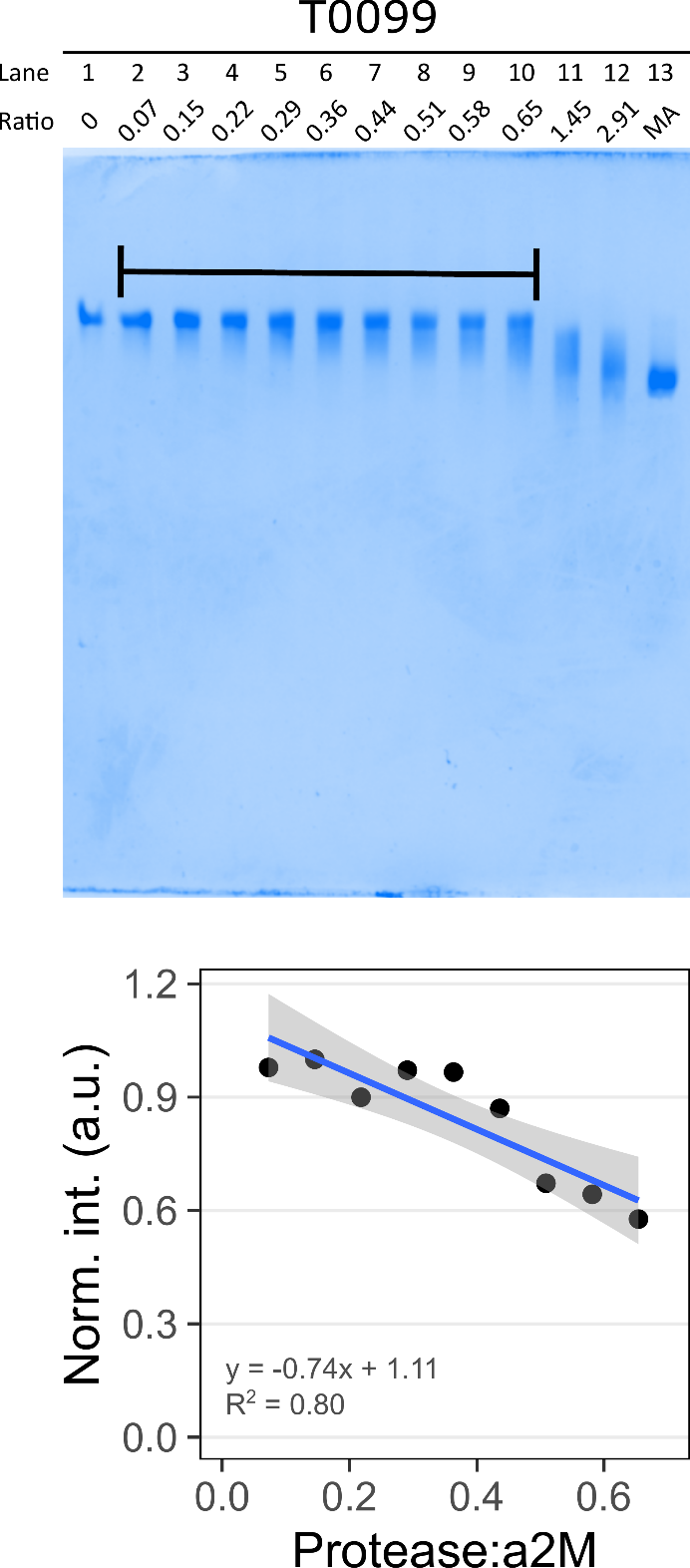


Figure S8. T0099 titration against α2M. Ratios indicate molar stoichiometry between protease and α2M. The molarity of P355 is based on the total protease concentration. The intensity was normalized to the band with no added protease (lane 1) for the given protease. The solid bars on the gels indicate the range used for linear regression (blue line) with the derived linear equation shown in the plot. Note that the data point from lane 1 was omitted in the regression as it was an outlier due a leak from the well. The grey area indicates the confidence interval (0.95) of the fit.


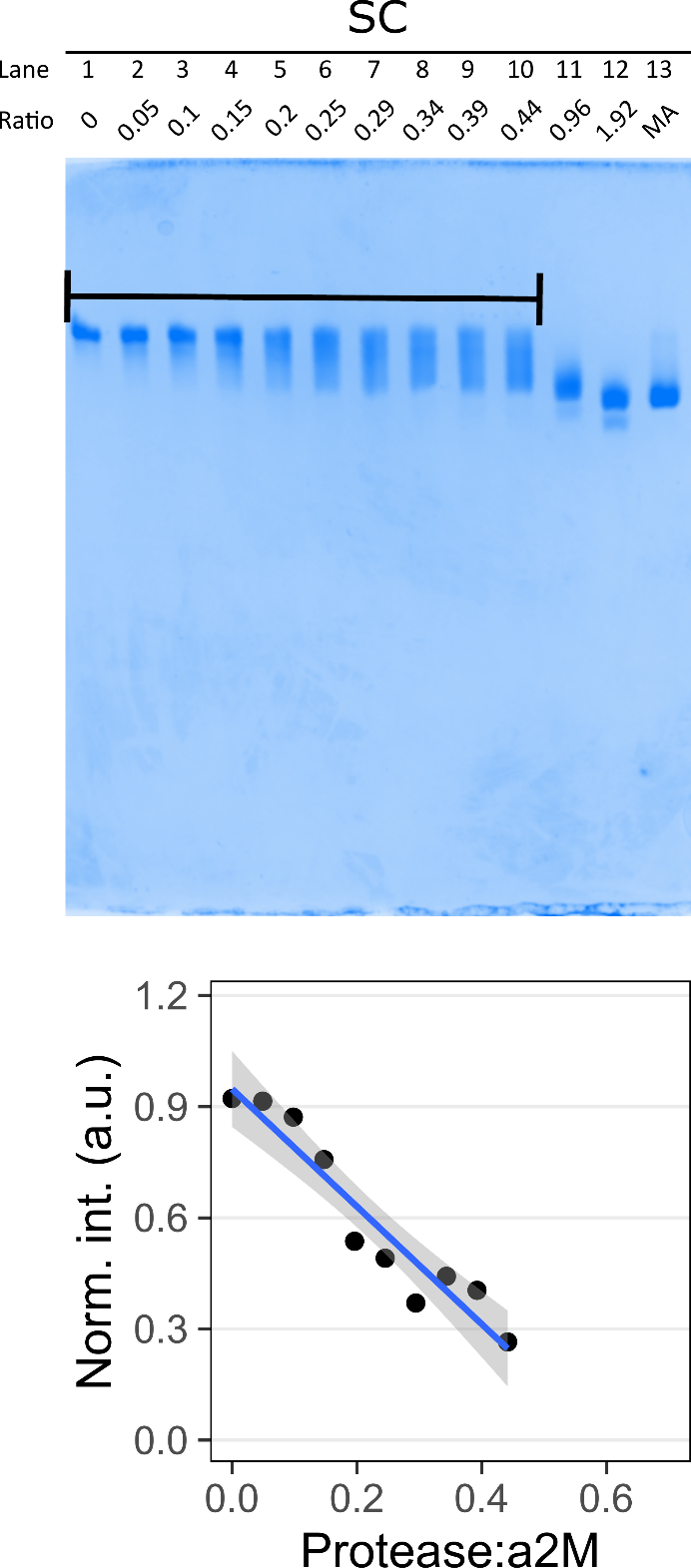


Figure S9. SC titration against α2M. Ratios indicate molar stoichiometry between protease and α2M. The molarity of P355 is based on the total protease concentration. The intensity was normalized to the band with no added protease (lane 1) for the given protease. The solid bars on the gels indicate the range used for linear regression (blue line) with the derived linear equation shown in the plot. The grey area indicates the confidence interval (0.95) of the fit.


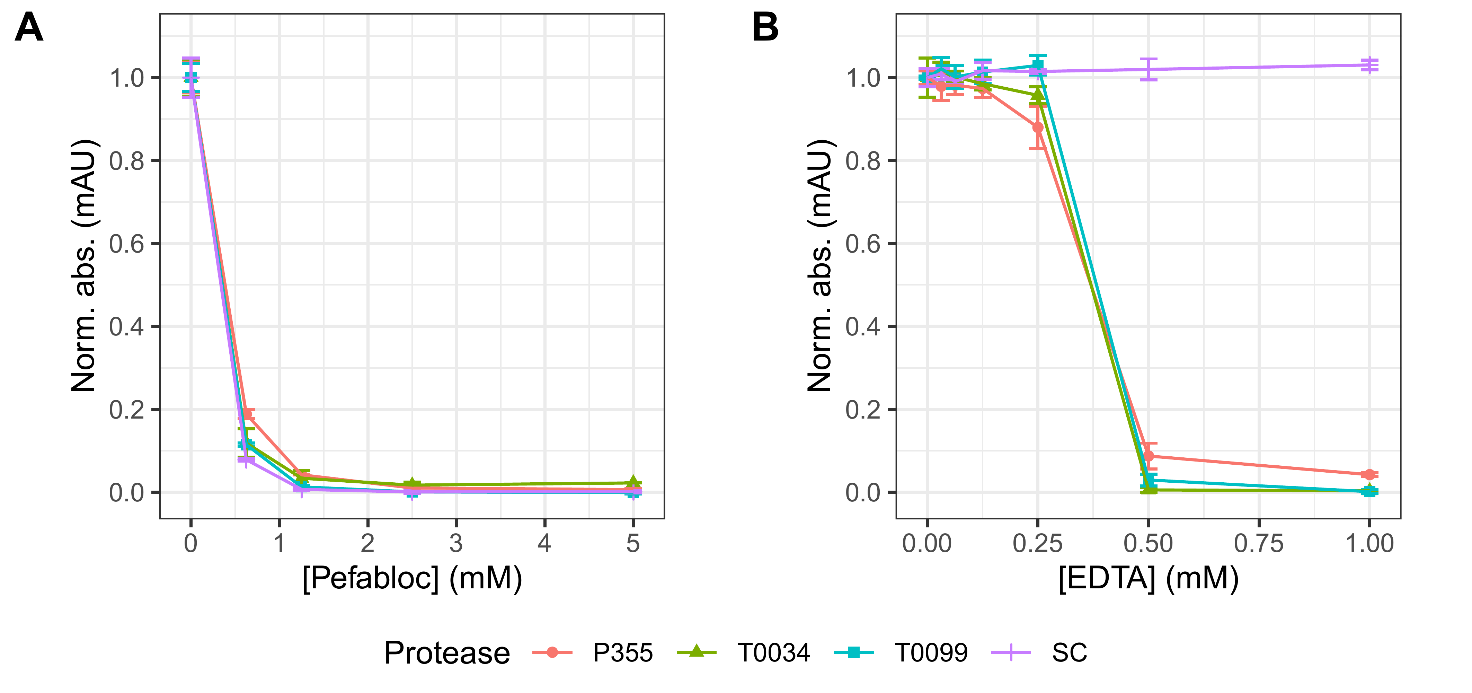


Figure S10. Inhibition with EDTA and Pefabloc®. **(A)** Matured proteases incubated with varying concentrations of EDTA (0-1 mM) for 1 at RT prior to the activity measurement. EDTA completely inhibited matured T0034 and T0099 activity at 1 mM, while minuscule activity remained for matured P355 at 1 mM EDTA. Matured SC was unaffected at all concentrations. The calcium concentration was 63 µM **(B)** Pefabloc® (0-5 mM) incubated with matured protease for 2.5 h. Every matured protease was completely inhibited at 2.5 mM. Activity using 0.2 mM AAPF was normalized to the highest activity for the given protease. Error bars show standard deviation (n = 3).


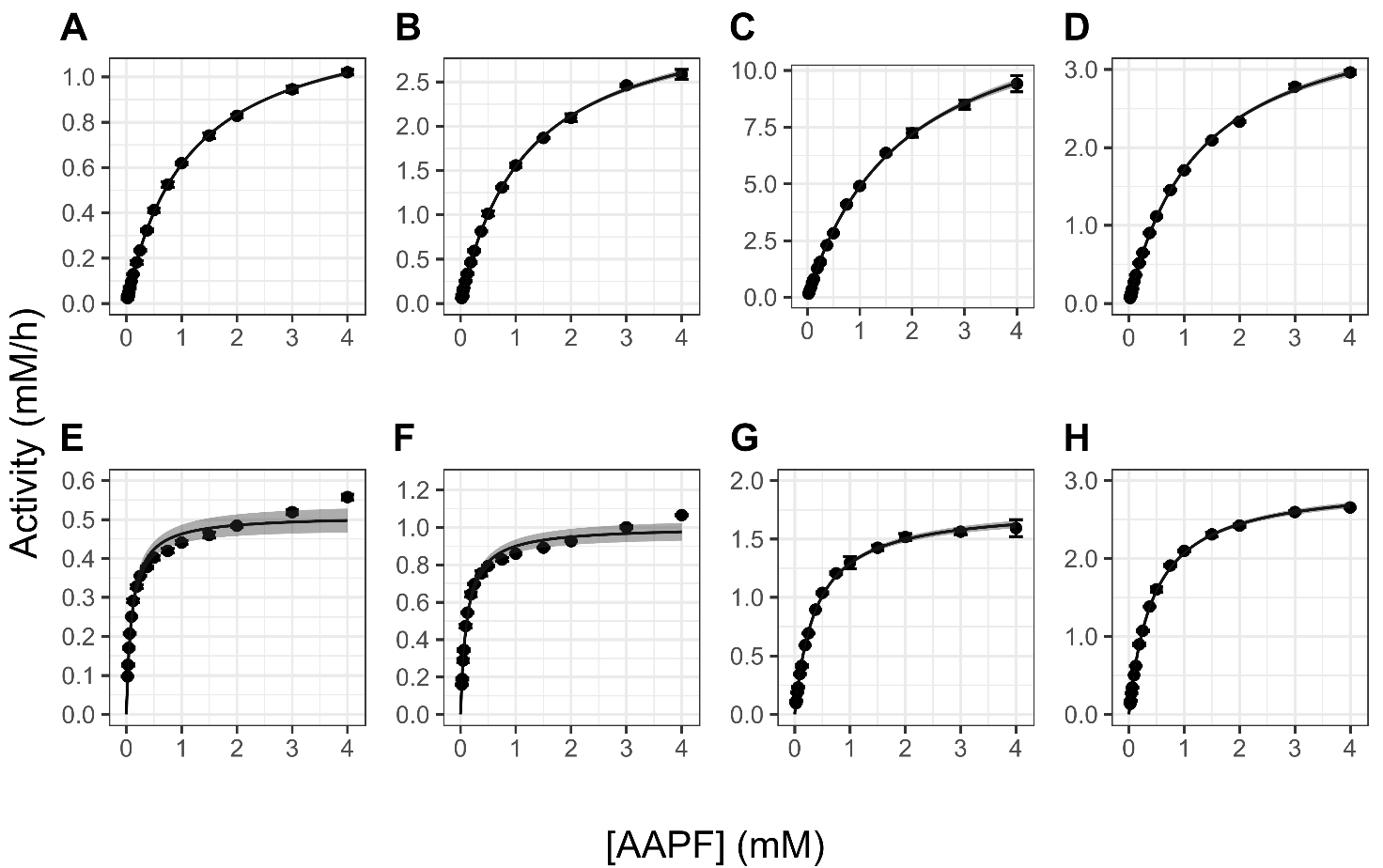


Figure S11. Michaelis-Menten kinetics at 25 and 45°C of matured proteases. Derived kinetic parameters are gathered in Table 1. The grey area indicates the confidence interval of 0.99. Error bars indicate standard deviation (n = 3). Note that the confidence interval for P355, T0034, and SC lies close to the fitted curve and error bars are within the markers in most cases.


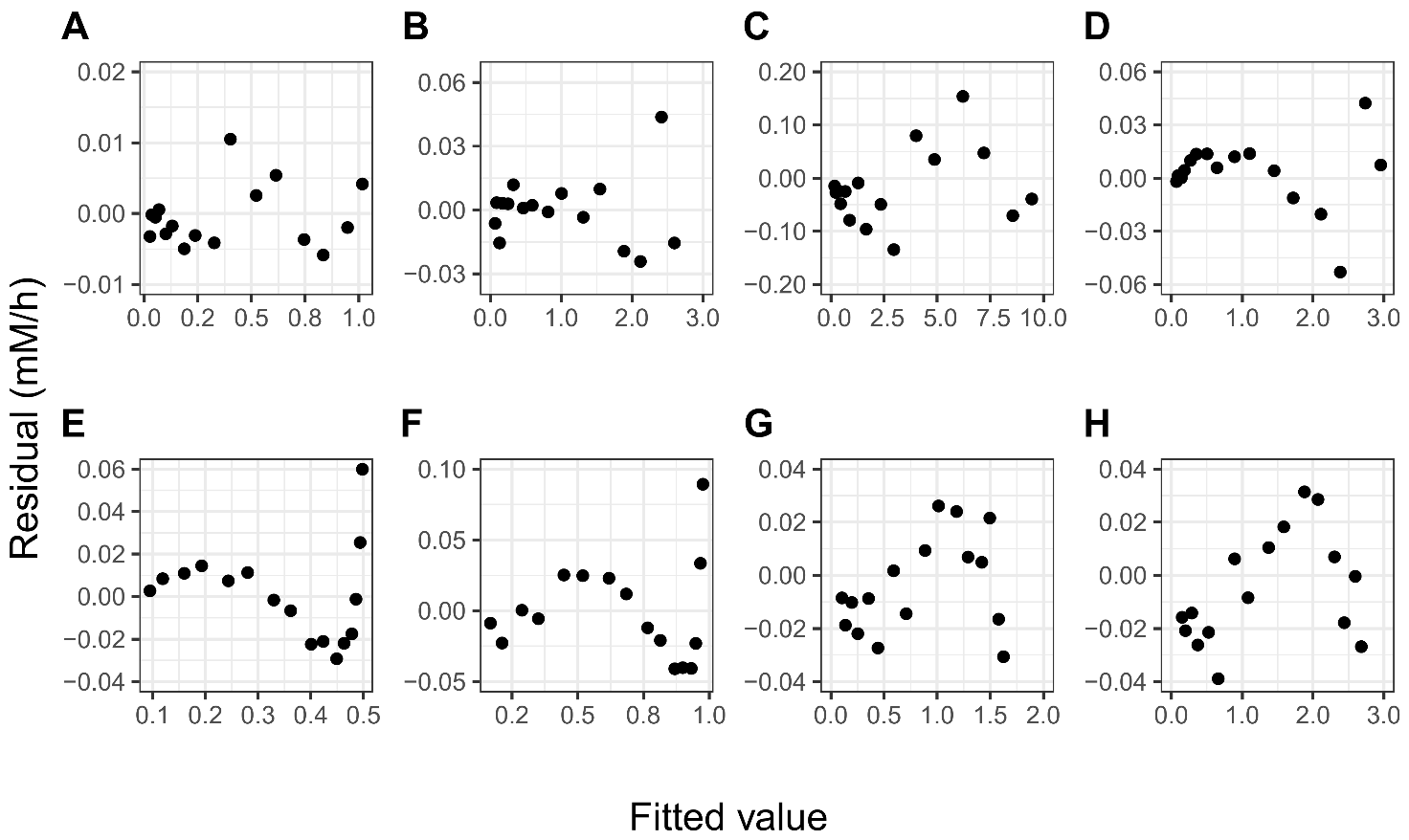


Figure S12. Residual plots from Michaelis-Menten fitting. Overall, low residual values and randomness are seen, which indicates good fits. However, some tendencies can be seen in panel D, E, and F, most prominent in E and F, indicating deviation from Michaelis-Menten kinetics.

Table S1. Statistics from the Michaelis-Menten fitting. Note that T0034 at T = 45°C was diluted by a factor 10 compared to T0034 at T = 25°C, thus explaining the lower V_max_. Estimate values are shown as means. Deviations show the confidence interval (0.99), RSE = residual standard error, n = 3.

| **Protease** | **T (°C)** | **RSE^1^** | **Parameters** | **Estimates^2^** |
| --- | --- | --- | --- | --- |
| P355 | 25 | 0.0045 | *V_max_* | 1.30 ± 0.02 |
|  | 25 |  | *K_M_* | 1.12 ± 0.04 |
|  | 45 | 0.0164 | *V_max_* | 3.36 ± 0.08 |
|  | 45 |  | *K_M_* | 1.17 ± 0.07 |
| T0034 | 25 | 0.0759 | *V_max_* | 13.77 ± 0.55 |
|  | 25 |  | *K_M_* | 1.83 ± 0.15 |
|  | 45 | 0.0208 | *V_max_* | 3.89 ± 0.11 |
|  | 45 |  | *K_M_* | 1.26 ± 0.08 |
| T0099 | 25 | 0.0231 | *V_max_* | 0.51 ± 0.03 |
|  | 25 |  | *K_M_* | 0.10 ± 0.03 |
|  | 45 | 0.0356 | *V_max_* | 1.00 ± 0.05 |
|  | 45 |  | *K_M_* | 0.12 ± 0.03 |
| SC | 25 | 0.0192 | *V_max_* | 1.78 ± 0.04 |
|  | 25 |  | *K_M_* | 0.38 ± 0.03 |
|  | 45 | 0.0223 | *V_max_* | 2.97 ± 0.06 |
|  | 45 |  | *K_M_* | 0.44 ± 0.03 |
| ^1^14 degrees of freedom.  ^2^Vmax is in mM/h and *K_M_* is in mM. | | | | |

# Data availability

The raw mass spectrometry data files have been deposited to the ProteomeXchange Consortium (http://proteomecentral.proteomexchange.org) via the PRIDE partner repository (Vizcaíno et al., 2015) with the dataset identifier PXD037979, along with the result file, that contains the identified peptides.

The remaining raw data are available upon request to Casper Bøjer Rasmussen ([cbr@mbg.au.dk](mailto:cbr@mbg.au.dk)).

# References

Barrett, A.J., Brown, M.A., and Sayers, C.A. (1979). The electrophoretically ‘slow’ and ‘fast’ forms of the α2-macroglobulin molecule. *Biochemical Journal* 181(2)**,** 401-418. doi: 10.1042/bj1810401.

Chase, T., and Shaw, E. (1967). p-Nitrophenyl-p′-guanidinobenzoate HCl: A new active site titrant for trypsin. *Biochemical and Biophysical Research Communications* 29(4)**,** 508-514. doi: <https://doi.org/10.1016/0006-291X(67)90513-X>.

Chase, T., and Shaw, E. (1969). Comparison of the esterase activities of trypsin, plasmin, and thrombin on guanidinobenzoate esters. Titration of the enzymes. *Biochemistry* 8(5)**,** 2212-2224. doi: 10.1021/bi00833a063.

Gettins, P., and Cunningham, L.W. (1986). Identification of proton resonances from the bait region of human .alpha.2-macroglobulin and effects of proteases and methylamine. *Biochemistry* 25(18)**,** 5011-5017. doi: 10.1021/bi00366a007.

Harwood, S.L., Diep, K., Nielsen, N.S., Jensen, K.T., and Enghild, J.J. (2022). The conformational change of the protease inhibitor α(2)-macroglobulin is triggered by the retraction of the cleaved bait region from a central channel. *J Biol Chem* 298(8)**,** 102230. doi: 10.1016/j.jbc.2022.102230.

Larsson, L.J., Neuenschwander, D.E., and Strickland, D.K. (1989). Reaction of proteinases with alpha 2-macroglobulin: evidence for alternate reaction pathways in the inhibition of trypsin. *Biochemistry* 28(19)**,** 7636-7643. doi: 10.1021/bi00445a020.

Manwell, C. (1977). A simplified electrophoretic system for determining molecular weights of proteins. *Biochemical Journal* 165(3)**,** 487-495. doi: 10.1042/bj1650487.

Schonbaum, G.R., Zerner, B., and Bender, M.L. (1961). The Spectrophotometric Determination of the Operational Normality of an α-Chymotrypsin Solution. *Journal of Biological Chemistry* 236(11)**,** 2930-2935. doi: <https://doi.org/10.1016/S0021-9258(19)76404-7>.

Sottrup-Jensen, L., Sand, O., Kristensen, L., and Fey, G.H. (1989). The α-Macroglobulin Bait Region: Sequence diversity and localization of cleavage sites for proteinases in five mammalian α-macroglobulins. *Journal of Biological Chemistry* 264(27)**,** 15781-15789. doi: <https://doi.org/10.1016/S0021-9258(18)71545-7>.

Vizcaíno, J.A., Csordas, A., del-Toro, N., Dianes, J.A., Griss, J., Lavidas, I., et al. (2015). 2016 update of the PRIDE database and its related tools. *Nucleic Acids Research* 44(D1)**,** D447-D456. doi: 10.1093/nar/gkv1145.
